# A Review of Graphene-Integrated Biosensors for Non-Invasive Biochemical Monitoring in Health Applications

**DOI:** 10.3390/s25216553

**Published:** 2025-10-24

**Authors:** Sourabhi Debnath, Tanmoy Debnath, Manoranjan Paul

**Affiliations:** 1 School of Computing, Mathematics and Engineering, Charles Sturt University, Bathurst, NSW 2795, Australia; sdebnath@csu.edu.au (S.D.); mpaul@csu.edu.au (M.P.); 2 Artificial Intelligence and Cyber Futures Institute, Charles Sturt University, Australia

**Keywords:** graphene, non-invasive sensors, biochemical sensors, biomedical applications, health monitoring, disease detection, cancer detection, tumour detection, glucose detection, drug detection

## Abstract

This review explores the transformative potential of graphene-based, non-invasive biochemical sensors in the context of real-time health monitoring and personalised medicine. Traditional diagnostic methods often involve invasive procedures that can be uncomfortable, pose risks, and limit the frequency of monitoring. In contrast, wearable sensors incorporating graphene offer a compelling alternative by enabling continuous, real-time tracking of physiological and biochemical signals with minimal intrusion. Graphene’s exceptional electrical conductivity, mechanical flexibility, biocompatibility, and high surface-area-to-volume ratio make it ideally suited for integration into skin-conformal sensor platforms. These properties not only enhance sensitivity and signal fidelity but also promote user comfort and long-term wearability, critical factors for the adoption of wearable health technologies. The discussion evaluates current developments in the design and deployment of graphene-based biosensors, with particular attention given to their role in managing chronic conditions, supporting preventative healthcare, and facilitating decentralised diagnostics. By bridging materials science and biomedical engineering, this review positions graphene as a key enabler in the shift towards more proactive, patient-centred healthcare models. The text also identifies ongoing challenges and future directions in sensor design, aiming to inform researchers working at the intersection of advanced materials and medical technology.

## 1. Introduction

Conventional methods for health monitoring and diagnosis often rely on bulky equipment and invasive procedures, such as blood sampling, which can cause discomfort or even pain for patients [1]. These approaches are often time-consuming and inconvenient. However, wearable biomedical sensors have garnered considerable attention in both research and commercial markets in 2025. The monitoring of human health is largely dependent on the collection of physical, chemical, and biological signals conveyed through the skin [2,3]. Wearable sensors offer a promising alternative by enabling the continuous and real-time detection of these signals with a high degree of accuracy [4,5,6]. When affixed to various parts of the body, these devices function as effective analytical tools [7,8,9]. The data acquired through wearable sensors plays a vital role in the diagnosis and treatment of a wide range of medical conditions [10,11,12,13]. In biomedical applications, such sensors are particularly valued for their sophisticated design, exceptional sensing performance for reliable biosignal detection, and superior mechanical flexibility, which allows seamless integration with the human body [14,15].

Owing to the mechanical mismatch between human skin and traditional rigid, silicon-based sensors, sensors should demonstrate mechanical flexibility to ensure optimal functionality [16]. In addition, a range of factors must be taken into account to support long-term, multifunctional, real-time, and cost-effective health monitoring systems that are both unobtrusive and user-friendly. These factors include biocompatibility, reliability, stability, comfort, convenience, miniaturisation, cost-efficiency, and resistance to biofouling [17]. Central to the performance of biomedical sensors is the careful selection of sensing materials. These materials must not only be responsive to biophysical or biochemical stimuli to enable accurate detection of health-related signals but also be compatible with the mechanical design of the sensor to allow for resilience against deformation during practical use [14].

Recent developments in wearable sensor technology have primarily focused on exploring various sensing materials. These include conductive polymers [18,19,20], carbon-based materials [18,19,20], metallic compounds, fluorescent dyes [21,22] and other advanced nanomaterials [23,24,25,26,27,28]. Various graphene-based materials—including pristine graphene (Gr), graphene oxide (GrO), and reduced graphene oxide (rGrO)—have attracted research interest. Graphene-based materials have found growing applications in wearable technologies due to their excellent electrical and thermal conductivity [29,30,31,32], large specific surface area [33,34,35], enhanced mechanical strength [36,37], and unique optical properties [38,39]. These attributes have improved overall performance and opened new possibilities for functionality in these devices. Integrating such advanced materials continues to drive innovation in this rapidly evolving field.

### 1.1. Comparative Mechanistic Attributes of Gr, GrO, and rGrO in Wearable Biosensing

Graphene offers several distinct advantages when utilised in wearable sensor technologies [17]. Its high specific surface area and atomic scale thickness ensure that every carbon atom in the ${\mathrm{sp}}^{2}$-bonded lattice is directly exposed to the sensing environment, maximising analyte interaction and coupling molecular binding events directly to the conduction pathway. This characteristic significantly enhances the sensitivity of graphene-based sensors, surpassing that of traditional silicon-based counterparts (e.g., silicon nanowires and silicene) [40,41,42,43].

In sweat sensing, ions and metabolites adsorb onto the graphene surface, inducing charge transfer near the Dirac point; the low density of states amplifies conductivity changes, enabling detection at physiological concentrations. In saliva sensing, the abundant oxygen-containing functional groups of GrO and rGrO enable dense covalent immobilisation of enzymes or aptamers, ensuring efficient electron transfer from bound analytes to the electrode. Tear sensing benefits from the hydrophilicity of GrO, which promotes rapid wetting and preconcentration of proteins and metabolites in microlitre volumes, enhancing sensitivity. In breath sensing, pristine graphene’s high carrier mobility and low density of states facilitate room temperature chemiresistive detection of volatile organic compounds (VOCs) via adsorption-induced Fermi level shifts, with nanoparticle decoration adding selectivity without compromising mobility. To consolidate these context-specific mechanisms, a comparative summary of the key physicochemical attributes of Gr, GrO, and rGrO is presented in Table 1. This highlights their differences in conductivity, functionalisation potential, mechanical and biocompatibility profiles, and most importantly, their suitability for particular biofluid sensing niches.

Beyond these context-specific comparisons, the inherent mechanical flexibility and ultrathin nature of graphene render it highly adaptable, allowing for intimate and conformal contact with biological tissues, including the skin [44,45] and eyes [46,47,48]. Such properties are particularly valuable in obtaining high-quality physiological signals, minimising the risks of irritation, motion artefacts, and contamination during monitoring [49]. In addition, graphene’s high optical transparency and electrical conductivity make it well-suited for bioimaging applications, facilitating the observation of biological tissues with minimal visual interference [50]. Its excellent electrical properties also promote efficient signal transmission and seamless integration with soft tissues, leading to a high signal-to-noise ratio (SNR) in the recording of electrophysiological signals [50]. The large specific surface area also supports efficient immobilisation of receptors such as enzymes, antibodies, and DNA, convenient functionalisation, and rapid electron transfer [43]. These attributes, combined with the flexibility to select between Gr, GrO, and rGrO to suit the physicochemical requirements of specific applications, distinguish graphene-based materials from other nanomaterials such as carbon nanotubes, metal oxides, and transition metal dichalcogenides [36,37,51,52,53]. They offer an unrivalled combination of sensitivity, adaptability, and integration potential for non-invasive biochemical sensing.

### 1.2. Complementary 2D Nanomaterials: MXenes, Silicene, and Biochar for Advanced Biosensing

In addition to graphene, other two-dimensional (2D) materials have recently attracted attention as potential substitutes or complements. MXenes, a large family of transition metal carbides and nitrides, possess metallic conductivity, hydrophilicity, and abundant surface terminations that enable rapid charge transfer and facile functionalisation. These features have been exploited in enzymatic, electrochemical, and hydrogel-based biosensors, demonstrating high sensitivity and mechanical compliance in wearable applications, although stability and large-scale fabrication remain open challenges [54,55]. Silicene, the silicon analogue of graphene, exhibits a buckled honeycomb lattice with tunable bandgap and high carrier mobility. Its structural compatibility with existing semiconductor technologies makes it particularly attractive for next-generation biosensors and nanoelectronic devices, with recent studies showing promising performance of doped silicene nanoribbons in biosensing [56,57]. Another material is biochar, a porous carbonaceous substance produced from biomass pyrolysis. Although structurally more heterogeneous than graphene, biochar offers low-cost, sustainable, and scalable production routes. Its abundant oxygen-containing surface groups facilitate bioreceptor immobilisation and electrochemical sensing, enabling recent biochar-based biosensors for clinical analytes such as interleukin-6 (IL-6) in inflammation and cancer monitoring [58]. Nevertheless, biochar generally exhibits lower conductivity, less structural uniformity, and more variable biocompatibility compared with graphene. Table 2 compares the distinctive properties of graphene, MXenes, silicene, and biochar, highlighting their potential roles in biosensor development.

Although these emerging materials show promise, graphene remains the most extensively investigated, offering a unique balance of conductivity, flexibility, biocompatibility, and technological maturity; hence, this review primarily focuses on graphene-based platforms while acknowledging complementary advances in other emerging nanomaterials.

**Figure 1:**
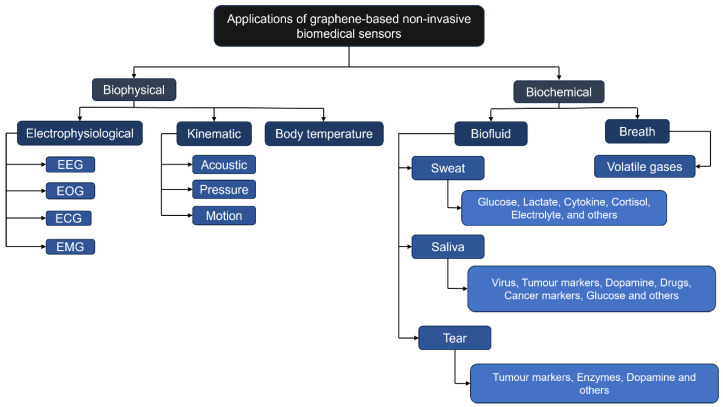
Applications of graphene-based non-invasive biomedical sensors for health monitoring. The schematic categorises sensor applications into biophysical and biochemical domains, detailing relevant biofluids and target analytes. It highlights graphene’s versatility in enabling multi-parameter health monitoring platforms, spanning electrophysiological signals to trace-level tumour markers, underscoring its transformative potential in personalised medicine.

The transformative potential of graphene lies in its adaptability across both biophysical and biochemical sensing domains, as illustrated in Figure 1. Its exceptional conductivity enables precise acquisition of electrophysiological signals such as ECG, EMG, and EEG, while its role as a transducer in biofluids like sweat, saliva, and tears supports the detection of diverse molecular biomarkers. This dual capability facilitates non-invasive monitoring of physiological parameters (e.g., heart rate, breathing rate, blood pressure, temperature) alongside biochemical indicators (e.g., glucose, stress hormones), thereby advancing health monitoring platforms that integrate functional status with metabolic and molecular data [17].

This review presents the recent advancements in graphene-based, non-invasive biochemical sensors for health monitoring over the past decade. Wearable biochemical sensors based on biofluids and exhaled breath show the potential for diagnosis systems [59,60]. Progress has been made in developing methods for identifying and monitoring various diseases by analysing biofluids such as blood, urine, sweat, breath, saliva, tears, and other bodily fluids. Patients with conditions like lung cancer, inflammatory lung disease, hepatic or renal dysfunction, and diabetes offer valuable insights through the analysis of their exhaled breath [61]. Advances have been made in identifying and measuring oxidative stress, as well as in its perioperative monitoring, by examining the composition of exhaled breath.

The principal contributions of this manuscript are as follows:

- Consolidates current knowledge and critically assesses the latest developments in the field of graphene-based, wearable biochemical sensors.
- Highlights the innovative potential of continuous, non-invasive biochemical monitoring for healthcare applications.
- Guides future research by addressing key technical and practical challenges.

**Figure 2:**
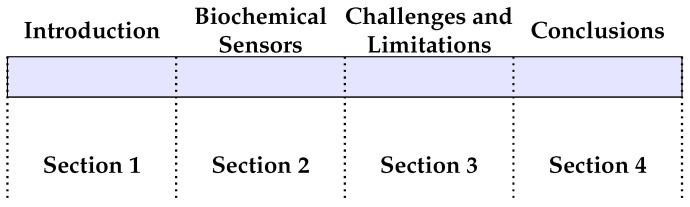
Structural roadmap of the present review, progressing from fundamental principles (Section 1) to sensor advancements (Section 2), followed by challenges (Section 3) and conclusions (Section 4). This framework first establishes context, then presents evidence, and finally synthesises findings to guide future research in an analytical and thematic manner.

This review is structured as outlined in Figure 2, progressing from foundational principles to applications (Section 2), followed by a critical evaluation of challenges and limitations (Section 3), and concluding with forward-looking perspectives (Section 4). The organisational framework emphasises the balance between performance gains and practical constraints, enabling not only a survey of the current landscape but also a synthesis of findings that informs future research and highlights the pathway from material properties to clinical translation.

## 2. Advancements in Biochemical Sensors

### 2.1. Biofluid-Based Sensing

According to the scientific literature, assessing current health conditions can be facilitated by examining biofluids secreted through human skin, such as sweat, which is considered the most readily accessible [62]. Saliva, sweat, and tears are biofluids that can be analysed to understand an individual’s health status. Sweat contains high levels of proteins, hormones, and DNA, making it an attractive option for non-invasive health monitoring [63,64]. Saliva has low concentrations of many biomolecules [64]. However, tears contain moderate levels of proteins and lipids, dominated by salts, and detecting protein levels can help identify syndromes such as dry eye [65] (see Figure 1; note how sweat, saliva, and tears each provide access to distinct but overlapping sets of biomarkers, necessitating fluid-specific sensor designs and functionalisation strategies).

#### 2.1.1. Sweat Biomarker Detection

Sweat eliminates excess nutrients, metabolic waste, and toxins [66]. Within the human body, three distinct types of sweat glands [67] contain various essential components, including metabolites such as lactate, glucose, urea, ethanol, and cortisol, as well as electrolytes like sodium, potassium, chloride, and ammonium. It also contains trace elements like zinc and copper, as well as a few large molecules, such as proteins, nucleic acids, neuropeptides, cytokines, and others [68]. Of these components, lactate and glucose are essential, as they are byproducts of glycolysis, usually absorbed by the liver [69]. In cases of liver damage, however, these metabolites can cause muscle fatigue and acidosis. Several research groups have documented that lactate concentration in sweat can serve as a valuable indicator of insufficient oxidative metabolism and pressure ischemia [70].

##### Glucose, Lactate, and Urea Detection

Type 1 diabetes results from inadequate insulin production in pancreatic beta cells, while Type 2 involves insulin resistance with relative insufficiency. Proper glucose level monitoring positively affects the treatment and improves the quality of the patient’s life [71,72]. The commonly known “finger pricking” method has a drawback that can lead to non-adherence. For instance, a patch-based electrochemical biosensor was developed for glucose monitoring in human sweat [73]. The device’s hybrid working electrode was constructed from electrochemically deposited gold-platinum alloy nanoparticles (AuPt NPs) on a rGrO surface, which was then integrated with a chitosan-glucose oxidase (GOx) composite. This configuration yielded an amperometric response with a sensitivity of 48 μ$\;\cdot \;{\mathrm{mM}}^{-1}$$\;\cdot \;{\mathrm{cm}}^{-1}$, a detection limit of 5 μM, and a response time of 20 s within a linear range of 0–2.4 mM. The authors assert that they intend to develop a wearable device for glucose monitoring based on sweat, which will incorporate sensors for pH, temperature, and humidity to enhance measurement accuracy. Furthermore, they aim to integrate this device into a complete system with a flexible printed circuit board.

**Figure 3:**
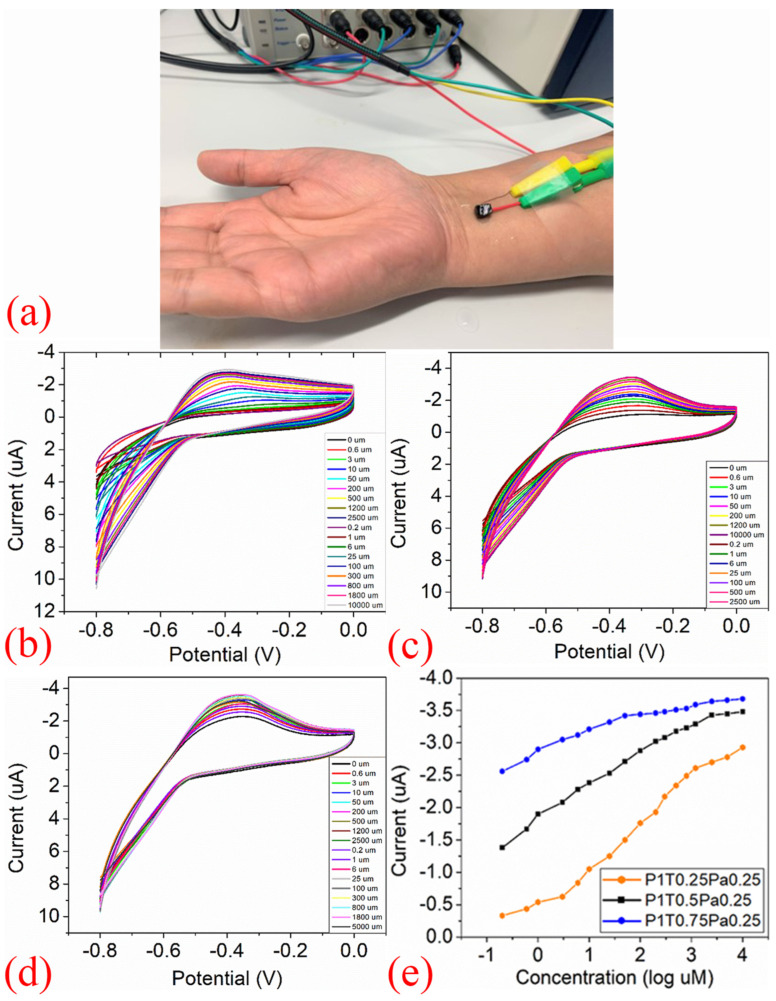
(**a**) Hydrogel-based wearable glucose sensing prototype on the arm. (**b**–**d**) Cyclic voltammetry (CV) curves of sensors with different coatings in varying glucose concentrations. (**e**) Logarithmic relationship between peak currents and glucose concentration. Together, these panels illustrate the amperometric biosensor principle, directly linking device design to dose-dependent electrochemical response, and demonstrate the transition from benchtop characterisation (**b**–**e**) to a viable wearable format (**a**). (Reproduced from [74] with permission from ScienceDirect).

A hybrid hydrogel nanocomposite sensor, composed of polyaniline (PANI) deposited on thermally exfoliated reduced graphene oxide (TEGrO) within a polyvinyl alcohol (PVA) matrix, was reported for glucose detection by Garg et al. [Figure 3]. The wearable prototype, positioned on the arm [Figure 3a], demonstrates practical deployment, while cyclic voltammetry (CV) scans [Figure 3b–d] reveal a systematic increase in peak currents with rising glucose concentrations, yielding the calibration curve in [Figure 3e]. This device achieved a wide linear detection range of 0.2μM to 10mM and a low detection limit of 0.2μM, highlighting its potential for non-invasive glucose monitoring [74]. The researchers report that their biosensor prototype achieves superior sensitivity for non-invasive glucose detection in sweat, highlighting the promise of hybrid hydrogel–graphene nanocomposites in wearable devices. By combining graphene’s conductivity with hydrogel’s biocompatibility, the hybridisation strategy enhances sensitivity, selectivity, and stability, though it also introduces fabrication complexity and potential reproducibility challenges. These advances point toward wearable platforms capable of continuous, wireless glucose monitoring that move beyond proof-of-concept toward practical, real-world use.

Furthermore, a wearable patch was developed using graphene doped with gold and a gold mesh to measure sweat-based glucose levels in individuals with diabetes. This device featured a serpentine bilayer of gold mesh with a glucose detection range of 10 μM–0.7 mM and a detection limit of 10 μM [75]. The researchers propose that the integration of their wearable devices with a portable and wireless power supply and data transmission unit would pave the way for point-of-care diabetes treatment. They emphasise the necessity for further research into transdermal drug delivery systems intended for human patients. The authors conclude that their advancements provide novel avenues for managing chronic diseases such as diabetes mellitus.

In 2018, Xuan et al. devised an economical and uncomplicated approach to produce highly flexible and conductive electrodes [76]. They employed a 3D porous structure composed of laser-induced graphene (LIG) and silver nanocomposites (AgNC). These electrodes demonstrated consistent and high electrical conductivity even under mechanical strain. Platinum and gold nanoparticles (PtAuNP) were incorporated into the 3D porous LIG structure to enhance the electrochemical properties, particularly for wearable glucose sensors. The resulting glucose sensor exhibited a low detection threshold of 5 μM, a suitable detection range from 0 to 1.1 mM (encompassing typical sweat glucose levels), and a high level of linearity (0.99). Additionally, the developed pH sensor has displayed a linear performance (66 mV$\;\cdot \;{\mathrm{pH}}^{-1}$) across a pH range of 4 to 7. It was emphasised that the stretchable and patternable electrodes were suitable for diverse applications in wearable electronics, including sensors and energy storage solutions. They further stated their intention to unify these devices with flexible printed circuit boards to realise a highly miniaturised smart healthcare monitoring system in the near future.

Lactate, a component of sweat, plays a crucial role in assessing patients’ physical condition and clinical diagnostics, ranking as one of the lowest molecular weight metabolites in sweat (lower than glucose) [77]. Khan et al. conducted a study in which they devised a hydrophobic wearable sensor from textiles to measure lactate [67]. The sensor, named Gr-PU-rGrO-PB, combines graphite-polyurethane (Gr-PU) with chemically treated rGrO and a phosphate buffer (PB) mediator to enhance electron transfer in electrode materials. The sensor featured an electrode system created using a template method, where both the working and counter electrodes were coated with the Gr-PU-rGrO-PB paste, and the reference electrode was filled with an Ag/AgCl solution. The electrode surface was coated with the Lactate Oxidase (LOx) enzyme to enhance sensitivity and catalytic efficiency. Conductive silver threads were stitched onto the textile base to establish the connectivity between the electrode tails and the device. The electrode’s ability was tested through cyclic voltammetry (CV) in a ferricyanide solution ranging from 5.0 mM to 25 mM, displaying a consistent linear response of anodic current for concentrations between 0.01 mM and 10.0 mM with relative standard deviations of approximately 0.2%. The electrode surface was also prepared with lactate oxidase and chitosan for amperometric and differential pulse voltammetric measurements. The sensor detected lactate levels in human sweat with a detection limit (LOD) of 0.4 mM and a quantification limit (LOQ) of 1.3 mM. Additionally, the sensor could sustain accurate measurements of lactate levels even after being washed in a household washing machine, with a relative standard deviation (RSD) of 3.06%. The scientists acknowledge a limitation in their research regarding the inability to validate and compare their results with blood lactate levels, an aspect they consider worthy of future investigation. They also envision that a similar methodology could be adapted for detecting a variety of metabolites, such as glucose, in alternative biofluids, including urine and saliva. Ultimately, the authors anticipate that their findings will catalyse fundamental research into wearable textile-based sensors for real-time healthcare monitoring and medical diagnostics.

However, there is a growing interest in detecting glucose and lactate simultaneously without relying on enzymes. Wang et al. [78] developed a method to create a metal–organic framework (MOF) electrode. They achieved this by using a flexible amino-functionalised graphene paper, modified through a simple interfacial synthesis process and an efficient dip-coating technique, to form a 2D-oriented assembly of ${\mathrm{Cu}}_{3}{\left(\mathrm{BTC}\right)}_{2}$ nanocubes. An important discovery was their ability to transfer 2D arrays of ${\mathrm{Cu}}_{3}{\left(\mathrm{BTC}\right)}_{2}$ nanotubes from oil–water interfaces onto amino-functionalised graphene paper, resulting in a tightly packed monolayer of uniformly sized ${\mathrm{Cu}}_{3}{\left(\mathrm{BTC}\right)}_{2}$ nanocubes on the paper electrode. The electrode exhibited sensing capabilities for detecting lactate and glucose in sweat, making it suitable for non-enzymatic electrochemical biosensing. Tests showed that the biosensor provided a linear dynamic range for lactate detection from 0.05 to 22.6 mM, with a detection threshold of 5 μM. It offered a range from 0.05 to 1775.5 μM for glucose detection, with a detection limit of 30 nM. Furthermore, another wearable sensor was developed to track glucose and lactate levels in human sweat non-invasively, incorporating a GrO and chitosan composite (GrO-Ch) on screen-printed electrodes (SPEs) modified with Prussian blue (PBl) to bind glucose oxidase (GOx) and lactate oxidase (LOx), resulting in a stable bio-receptor deposition on the electrochemical platform. The analytical performance of the sensors was defined by flow injection analysis (FIA). From FIA, the LOD of the sensors for glucose and lactate were found to be 6.7 mM and 28 mM, respectively [79]. Wang et al. [78] express their intent to refine existing conditions to establish a calibration curve for the direct detection of sweat in forthcoming studies. They note that the subsequent step in advancing wearable sweat analysis devices will involve the implementation of a wireless system incorporating a miniature integrated circuit. The authors foresee that the broader applications of their strategy will facilitate the development of minimally invasive diagnostic tools, non-enzymatic instruments, and wearable medical bionanoelectronics, while their modular approach is expected to yield new insights into the design of metal–organic framework-based electrodes for biosensing applications and wearable electronics.

Urea is a small molecule that serves as the primary end product of both internal and external protein metabolism. It is uncharged and lipid-soluble, allowing it to easily permeate biological membranes and distribute evenly throughout the body’s water. Monitoring urea levels can indicate kidney function [80]. It was reported that many individuals with diabetes also suffered from kidney disease [81,82]. Therefore, it is essential to monitor urea in conjunction with glucose. Furthermore, the simultaneous tracking of glucose and urea can reduce the time and expense associated with clinical testing.

Promphet et al. designed a tailor-made sensor using cotton thread to detect glucose and urea in human sweat [83]. They enhanced the cotton thread surfaces with cellulose nanofiber/chitosan-graphene oxide, which improved enzyme immobilisation efficiency and overall sensor performance. For glucose, the sensor had a linear range of 0.1–3 mM with a detection limit of 0.1 mM and for urea, a linear range of 30–180 mM with a detection limit of 30 mM. This colourimetric sensor employs a structurally adaptable design that could detect glucose and urea in human sweat, differentiating between physiologically typical and atypical concentrations. Its flexibility allows it to integrate into clothing and accessories. The researchers highlight that the flexible characteristics of their sensor facilitate seamless integration with apparel and accessories, allowing for real-time and continuous monitoring of diabetes and kidney failure through analysis of the wearer’s sweat. They propose that this innovative platform may pave the way for blood-free diagnostic solutions in healthcare applications.

##### Cortisol and Cytokines Detection

The human body relies on endocrine glands to secrete hormones that regulate and control specific cells and organs [84,85]. Hormone imbalances can lead to diseases, including cardiovascular disease, hyperplasia, adenoma, osteoporosis, and cancer [86]. Detecting hormones through human sweat is a biomarker tool to reveal cortisol secretion due to emotional and psychological stress [87,88]. Additionally, autoimmune diseases can be detected by measuring vital biomarkers like tumour necrosis factor-alpha (TNF-α), produced by activating monocytes/macrophages in the inflammatory response [89,90,91].

**Figure 4:**
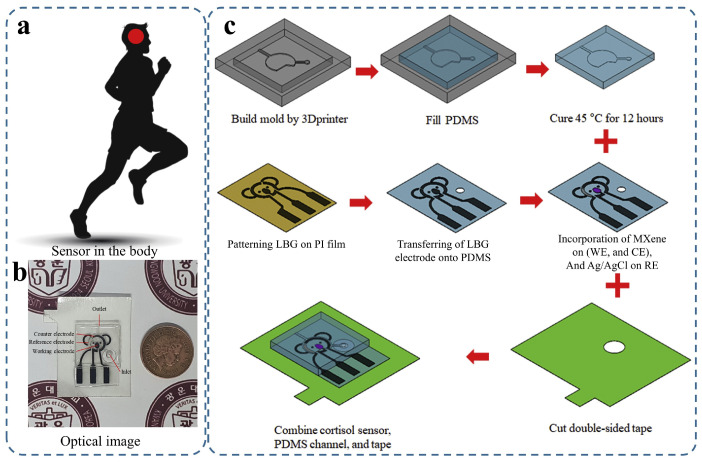
Wearable patch sensor for cortisol and its fabrication protocol. (**a**) Placement of the cortisol-sensing patch on the human torso. (**b**) Optical micrograph of the fabricated patch. (**c**) Stepwise fabrication protocol with microfluidic integration. The use of microfluidics is critical for handling low-volume biofluids like sweat, ensuring reliable sample delivery to the sensor surface. This figure highlights how graphene’s conductivity, combined with advanced material integration (LBG, MXene, PDMS), enables a functional, flexible wearable device for stress biomarker monitoring, while raising considerations of stability and user comfort. (Reproduced from [92] with permission from ScienceDirect).

In 2021, Nah et al. [92] reported a portable electrochemical impedimetric immunosensor for non-invasive cortisol detection in sweat, realised in a wearable patch format [Figure 4]. The practical deployment of the device is shown in situ on the human torso [Figure 4a], while the fabricated patch is visualised through an optical micrograph [Figure 4b]. The detailed fabrication protocol [Figure 4c] highlights the engineering sophistication required to integrate a ${\mathrm{Ti}}_{3}C_{2}T_{x}$ MXene–loaded laser-burned graphene (LBG) electrode network within a PDMS substrate and overcome the disconnection issue between graphene flakes. This architecture, incorporating a 3D-printed microfluidic module, enabled a detection range of 0.01–100 nM with a detection limit of 88 pM, demonstrating suitability for point-of-care hormone monitoring. These panels illustrate the sensor’s transition from benchtop prototype to a microfluidic-integrated wearable, exemplifying how graphene-enabled composites can bridge molecular sensitivity with practical device design, while also underscoring the remaining challenges of long-term stability and user comfort. Furthermore, the authors mention that this patch can be integrated with a wearable electrochemical front-end, enabling impedance signal monitoring and wireless data transfer for diagnostic purposes via smartphones.

Moreover, in 2020, Torrente-Rodríguez et al. studied the behaviour of cortisol using a five-electrode graphene-based sweat stress sensing system (GrS4) fabricated on a polyimide (PI) substrate, an integrated wireless mental health (mHealth) device [93]. Their device’s detection limit, defined as the concentration of cortisol that leads to a 10% decrease in signal, is 0.08 ng$\;\cdot \;{\mathrm{mL}}^{-1}$. The range of concentrations for 20–80% inhibition of the enzymatic tracer was 0.43–50.2 ng$\;\cdot \;{\mathrm{mL}}^{-1}$ cortisol. Additionally, cytokines are soluble proteins or glycoproteins outside cells that play crucial roles as intercellular regulators and mobilisers for cells engaged in innate and adaptive immunity [94] in controlling the survival/death of cells, their growth and differentiation, as well as the effector functions in tissues and immune cells [95]. Cytokines influence nearly every biological function, including embryonic development, disease onset, the general immune response to infections, targeted responses to specific antigens, changes in cognitive abilities, and the progression of age-related degenerative conditions [94]. The authors suggest that their work has the potential to transform paradigms in clinical research and mental health monitoring in the near future. They propose that continuous dynamic profiling of stress responses through sweat sensors presents new opportunities for fundamental research and for daily mental health assessments. The authors also indicate that their integrated sensing approach could lead to technological enhancements in personalised human performance management and mental health, ultimately contributing to decentralised healthcare surveillance at the metabolic level.

In 2020, Wang et al. developed a flexible, renewable biosensor using a graphene-Nafion field-effect transistor (GNFET) to detect cytokine storm biomarkers in human biofluids without the need for dilution [96]. This composite film minimised nonspecific adsorption and enabled the biosensor to be regenerated. The device detected cytokines such as IFN-γ, an inflammatory and cancer biomarker, with a detection range between 0.015 and 250 nM and LOD down to 740 fM in undiluted human sweat. The biosensor also exhibited no visible mechanical damage and maintained consistent sensing and crumpling responses for tests of 80 and 100 cycles, respectively. The work projects that the biosensor is poised to open avenues for developing wearable biosensing systems capable of distinguishing patients with acute infectious diseases and monitoring their health in everyday life. In particular, they propose that their biosensor, once modified with specific probes for the COVID-19 biomarker, could facilitate rapid and convenient monitoring of this infection in human biofluids. They suggest that such a biosensor could play an important role in aiding hospitals in stratifying COVID-19 patients based on disease severity.

##### Electrolytes Detection

Sweat consists of sodium (${\mathrm{Na}}^{+}$) and chloride (${\mathrm{Cl}}^{-}$) ions among all electrolytes, as well as metabolites and hormones. Therefore, it serves as a valuable source of information for health monitoring purposes [97]. Researchers found that changes to the cystic fibrosis transmembrane conductance regulator (CFTR) protein can affect the movement of ${\mathrm{Na}}^{+}$ and ${\mathrm{Cl}}^{-}$ in fluid-secreting cells [98,99,100], resulting in conditions such as dehydration, hyponatremia, and hypokalemia [101,102] due to fluctuations in sweat ion concentrations.

Park et al. used graphene-based ink to create a conductive, stretchable, and printable wearable biosensor that can measure sodium ion concentration in sweat during physical activity. They achieved this by employing a controlled exfoliation and mixing process guided by fluid dynamics. The screen-printed exfoliated graphene flakes (EGrFs) conductors demonstrated high electrical conductivity and mechanical properties. Incorporating these conductors, they created a stretchable electrochemical sensor for sodium ions with serpentine-structured electrodes. This biosensor maintained its performance under considerable strain and accurately measured sodium ion levels ranging from $10^{-1}$ to $10^{-4}$ M, with a sensitivity threshold of $2.5\times 10^{-6}\;M$ [103]. The work concludes that their functional graphene inks present substantial potential for various flexible wearable electronic applications. Their stretchable and conductive inks, produced through a scalable process, can support mass production of wearable devices.

Hua et al. introduced a wearable sensor to track ammonium (${\mathrm{NH}}_{4}^{+}$) levels in sweat. This sensor employed a specific ionophore in its ion-selective membrane (ISM). It is incorporated into a three-dimensional porous structure of a metal–organic framework/graphene (MOF/Gr) composite. The device demonstrated a Nernstian response of 59.23 ± 1.52 mV/log [${\mathrm{NH}}_{4}^{+}$] (with a 4.72% relative standard deviation based on four measurements), highlighting its high sensitivity. Furthermore, the sensor exhibited minimal resistance to charge transfer. The hydrophobic properties of the MOF/Gr layer ensure a consistent and reversible response to varying ${\mathrm{NH}}_{4}^{+}$ levels by preventing the formation of a water layer at the interface between the ISM and the screen-printed carbon electrodes. The research found that the sensor’s ${\mathrm{NH}}_{4}^{+}$ ion detection range lies between $10^{-6}$ and $10^{-1}$ M [104].

**Figure 5:**
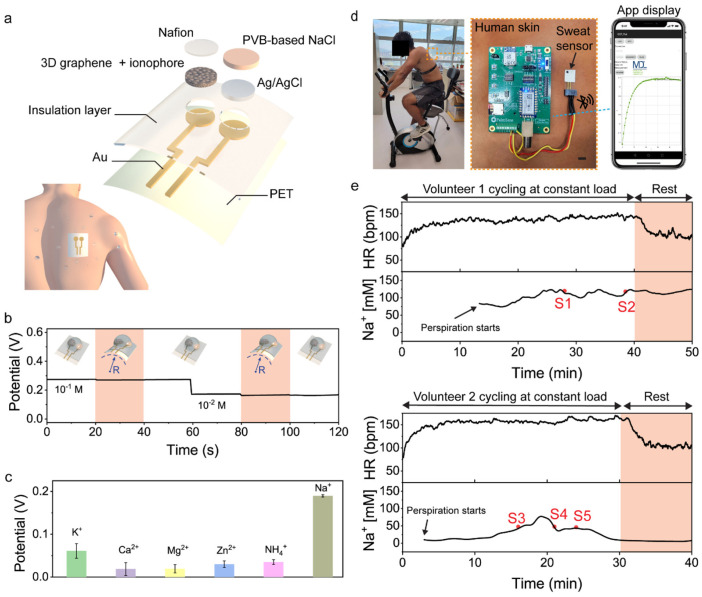
Flexible ${\mathrm{Na}}^{+}$ sweat sensor system and performance validation. (**a**) Layer schematic of the sensor. (**b**) Signal stability under bending. (**c**) Selectivity for ${\mathrm{Na}}^{+}$. (**d**) On-body cycling test with wireless prototype. (**e**) Real-time ${\mathrm{Na}}^{+}$ and heart rate monitoring. (Reproduced from [104] with permission from ACS Publications). This figure demonstrates a full validation pathway, integrating design, mechanical robustness, analytical specificity, and on-body performance. The real-time data (**d**,**e**) confirm the sensor’s capability to correlate biochemical and physiological metrics, establishing a benchmark for wearable biosensor maturity beyond sensitivity or LOD. (Reproduced from [105] with permission from ACS Publications).

Wearable sweat sensors offer significant potential for personalised healthcare and athletic monitoring, yet their performance is often hindered by water layer formation on ion-selective electrodes, reducing sensitivity and stability. Yeung et al. [105] addressed this by employing chemical vapour deposition to produce three-dimensional graphene electrodes with a gradient porous structure. This architecture suppressed water layer formation, enhanced ion transport, and expanded the electroactive surface, enabling sensitive, selective, real-time ${\mathrm{Na}}^{+}$ detection. Figure 5 summarises validation: the 3D framework Figure 5a demonstrated mechanical robustness under bending Figure 5b, strong ${\mathrm{Na}}^{+}$ selectivity over $K^{+}$ and ${\mathrm{Ca}}^{2+}$ Figure 5c, and reliable on-body cycling performance with wireless transmission (Figure 5d,e). Quantitatively, the design achieved a seven-fold increase in electroactive area and a sensitivity of 65.1 ± 0.25 mV per decade (n = 3, RSD = 0.39%). The authors suggest that their sensor could be extended to measure additional ions, enabling multiplexed health monitoring. However, inter-individual variability highlights the need for reproducible fabrication and large-scale validation, underscoring the gap between laboratory optimisation and practical deployment. Collectively, these findings demonstrate highly sensitive, real-time detection and establish a foundation for reliable non-invasive sweat sensing.

Additionally, An et al. proposed a flexible, paper-based, ion-selective electrode (ISE) for real-time sweat analysis [106]. The researchers enhanced the super-hydrophobic paper matrix with a fluorinated alkyl silane to reduce the water-layer effect. They employed a high-quality graphene suspension with high conductivity and capacitance as an ion-to-electron transducer to further enhance the stability of the potential. Their solid ISE features four channels for simultaneous measurement of K^+^, Na^+^, Cl^−^, and pH. The authors conveyed that their ISE sensor system can be improved by developing a micro- or nano-scale sensor chip capable of simultaneous ion testing. They envisage integration with self-powered units and mobile devices for in situ analysis, highlighting its utility in healthcare settings.

##### L-Cysteine Detection

Huang et al. proposed a transparent and flexible graphene-based field-effect transistor (GFET) wearable nanosensor to detect biomarkers in bodily fluids [107]. The binding between the receptor and biomarkers in bodily fluids resulted in a measurable shift in graphene’s carrier density, which was used to quantify the levels of these biomarkers. The nanosensor was reported to maintain its structural integrity and consistent electrical performance even after undergoing 100 deformation cycles, including bending at 175 μm radii, folding at 150 °C, and shrinking by 50%. The effectiveness of this device was demonstrated by its ability to detect L-cysteine, an amino acid associated with various health conditions, in concentrations ranging from 0 to 4800 × $10^{-6}$ M in pure human sweat, with a detection threshold of 0.022 × $10^{-6}$ M. The authors assert that their ultra-flexible and transparent graphene field-effect transistor wearable nanosensor is promising for medical applications, with potential use in contact lenses. They anticipate extending the platform for detecting additional biomarkers through proper receptor functionalisation.

The performance of these advanced sensors is further illustrated in Table 3 and the following figures. Figure 3 demonstrates a hydrogel-based glucose sensor, showcasing its practical on-arm deployment and the critical calibration data (CV curves and log-linear response) that underpin its reliable operation. Figure 4 details a sophisticated microfluidic-integrated patch for cortisol sensing, highlighting the intricate fabrication process and the device’s placement for stress hormone monitoring. Furthermore, Figure 5 presents a robust sodium ion sensor, providing evidence of its mechanical resilience (b), exceptional selectivity (c), and successful validation through real-time on-body trials with wireless data transmission (d,e).

To enable a rigorous, comparative assessment of sweat-based graphene biosensors, we establish biofluid-specific benchmarks tailored to physiological sweat dynamics. These parameters assess device readiness for translation into practical health and fitness applications. As detailed in Table 3, each sensor was evaluated against these criteria:

1. Sensitivity: A signal change of ≥10% across the detection range to ensure the signal is clearly distinguishable from noise.
2. Detection Range: Coverage of clinically relevant concentrations for the target analyte in sweat (e.g., 0.01–25 mM for metabolites—lactate/glucose, 10–100 mM for electrolytes).
3. Limit of Detection (LOD): A threshold of <10 μM, necessary for detecting baseline levels of key metabolites like glucose.
4. Response Time: A rapid response of <60 s, which is important for tracking dynamic physiological changes during exertion.
5. Durability: Demonstrated stability for >8 h of continuous use or >100 deformation/use cycles, reflecting the need for robustness during a typical activity session.

All unknown (“U”) values were conservatively treated as not meeting benchmarks.

The benchmark analysis of sweat-based graphene biosensors reveals an advancing yet still maturing field wherein few devices reconcile clinical-range sensitivity, rapid kinetics, and operational stability within a wearable format. Most sensors achieve moderate success (scores of 2–3 out of 5), demonstrating commendable but incomplete adherence to the multidimensional performance criteria necessary for robust real-world utility. Only four of sixteen systems, the ${\mathrm{NH}}_{2}$GP/${\mathrm{Cu}}_{3}{\left(\mathrm{BTC}\right)}_{2}$, the AuPt NPs/rGrO/chitosan-${\mathrm{GO}}_{x}$, AgNC/3D LIGr/PtAuNP, and the EGrFs/TPU, NMP sensors, met all five benchmarks. While potentiometric sodium sensors exhibit excellent sensitivity and relevant detection ranges, their LODs remain insufficiently low for trace biomarker applications, revealing a specificity challenge. Conversely, certain affinity-based sensors, such as those targeting cortisol and cytokines, excel in LOD but often compromise on response speed and long-term durability, highlighting an intrinsic trade-off between analytical sensitivity and kinetic responsiveness. A pervasive issue within the literature is inconsistent reporting of response times and durability under physiologically relevant conditions, which affects direct comparison and emphasises a gap between laboratory validation and practical deployment. These findings underscore the critical need for standardised testing protocols that encompass reliability, reproducibility, and user-centric operating environments, guiding the transition of promising prototypes toward commercial-ready, wearable biosensors capable of delivering consistent, real-time physiological monitoring.

#### 2.1.2. Saliva Biomarker Detection

Saliva is a viable alternative to blood analysis since it correlates with blood analytes [108]. It plays a vital role in biochemical analysis for detecting various analytes as a non-invasive medium for biomarker discovery. It contains biomarkers, including microbes, antibodies, DNA, ribonucleic acid (RNA), metabolites, lipids, and proteins [109], which can identify multiple diseases [110,111]. These biomarkers can be produced by inflammatory cells, immune system molecules, and other sources [112,113]. The pre-treatment of saliva often involves centrifugation to remove large particles and food remnants, which do not contribute to the analytical outcome. Following a dilution step, the literature suggests the most effective 1:10 dilution ratio for human saliva samples. Furthermore, spiking saliva samples with known quantities of analytes is observed to standardise the detection process.

Saliva-based biosensors face several complex practical challenges that require careful consideration, including the low concentration of biomarkers in saliva compared to blood and the need to ensure sample consistency and stability, essential for achieving continuous and reliable monitoring without significant error. Consequently, developing highly sensitive detection systems through focused research and innovation is essential. Saliva collection is relatively simple compared to other bodily fluids, but there is a risk of contamination from food and drink. In such cases, biosensors with immobilised receptors and high specificity for the target analyte should be used [61]. Mouth injuries and gum bleeding can also contaminate saliva analyte sensing. Biofouling is another issue that salivary-based biosensors may encounter, which occurs when various saliva contaminants, such as food residues and oral cavity bacteria, accumulate on the transducer surface through non-specific interactions, thereby affecting the biosensor’s performance. A protective coating around the biosensor could reduce biofouling issues. Developing oral-cavity biosensors capable of detecting multiple analytes will advance the use of saliva-based wearable devices.

The storage conditions of saliva samples are also crucial, as they influence the stability of analytes and the reliability of the analysis [114]. Despite the existence of sophisticated laboratory methodologies for saliva analysis, such as immunoassays (including radioimmunoassay, chemiluminescence immunoassay, and electrophoretic immunoassay), DNA-based techniques, salivary proteomics, and the prevalent Enzyme-Linked Immunosorbent Assay (ELISA), these approaches are centralised, time-consuming, and financially demanding. They require extensive sample preparation, data acquisition, and sample shipment alongside specialised personnel. One of the challenges is the sensitivity of the target recognition layer to variations in pH and temperature, which can negatively affect its functionality. Biofouling on the sensor surface can also reduce analytical precision. Though the sensors for salivary analysis pose several challenges regarding accuracy, reliability and reproducibility, researchers focus on developing biosensors to facilitate prompt, cost-effective, and efficient diagnostic procedures.

Incorporating graphene in saliva-sensing biosensors may offer several advantages over other materials, such as improved mechanical stability and flexibility, allowing biosensors to withstand the stress caused by mouth movements during talking, eating, and drinking. Different research groups reported that graphene-based saliva sensors are promising tools for detecting health conditions, as presented below.

##### Coronavirus Detection

Samavati et al. presented an approach to monitoring the COVID-19 virus using patients’ saliva and characterise their work as a promising diagnostic tool, highlighting the advantage of remote operation capability [115]. The study introduced a gold-fibre Bragg grating (Au/FBG) probe, augmented with GrO, to detect varying concentrations of COVID-19 in salivary samples. They analysed the salivary specimens of six COVID-19 patients, whose ages ranged from 34 to 72 years, with a median age of 58.5. The group contained two females and four male participants. The probe was used to predict shifts in both the wavelength and intensity of light when immersed in patients’ saliva. Deviations from the baseline established with healthy saliva indicated the presence of the virus, confirming infection. For a patient undergoing the hyperinflammatory phase of COVID-19, with a viral load quantified at $1.2\times 10^{8}$ copies$\;\cdot \;{\mathrm{mL}}^{-1}$ in their saliva, the probe exhibited a maximum wavelength shift of 1.12 nm and an intensity variation of 2.01 dB after 1600 s. However, for a patient in the initial phase of infection, characterised by a viral concentration of $1.6\times 10^{3}$ copies$\;\cdot \;{\mathrm{mL}}^{-1}$, the observed changes were 0.98 nm for wavelength shift and 1.32 dB for intensity change. Early detection efforts, conducted within 10 s of exposure to saliva from a patient at an early stage of infection, yielded modulation in the detected light’s wavelength and intensity of 0.39 nm and 0.49 dB, respectively. It was reported that the deviation from the baseline wavelength and intensity of healthy saliva increased beyond 200 s, reaching a plateau. The authors propose further development towards transforming the laboratory sensor into a deployable device for pandemic control.

For an electronic approach, Ban et al. developed a label-free GFET biosensor functionalised with DNA aptamers to detect SARS-CoV-2 proteins and viral particles directly in saliva [116]. They assert that their platform could facilitate accessible point-of-care diagnostics. The handheld, wireless device quantified spike (S) and nucleocapsid (N) proteins with detection limits of 1.28 and 1.45 plaque-forming units (PFU)/mL, respectively, providing results within 20 min. The sensor also demonstrated a considerable response to key concern variants, including Omicron (B.1.1.529), within femtomolar to nanomolar concentration ranges, while exhibiting a marked decrease in response to other viral particles and proteins. It was reported that the GFET sensor’s readings were consistent with those of Reverse Transcription Polymerase Chain Reaction (RT-PCR) and effectively differentiated between positive and negative clinical saliva samples during the surge of the Delta and Omicron variants. Although the GFET sensor’s positivity rate showed reduced sensitivity in the later stages of the pandemic, it was statistically in agreement with population-scale epidemiological data, demonstrating its high precision in detecting clinically and epidemiologically relevant variants of SARS-CoV-2. Increased levels of cytokines such as interferon (IFN), interleukin (IL), and tumour necrosis factor (TNF) are essential markers for recognising COVID-19 patients at risk of progressing to severe or critical conditions [117]. Therefore, developing sensors to track these biomarkers in patients is crucial for enabling early detection and treatment of those at risk of deterioration. Hao et al. introduced a biosensing device called an aptameric dual-channel graphene-TWEEN 80 field-effect transistor (DGTFET), which can accurately detect IFN-γ, TNF-α, and IL-6 within seven minutes. The biosensor detected these cytokines at deficient concentrations ($476\times 10^{-15}$, $608\times 10^{-15}$, and $611\times 10^{-15}$ M) in various biofluids, including serum, saliva, urine, and sweat. The DGTFET biosensor incorporated a sensing channel coated with a specific cytokine aptamer on graphene for target detection and a reference channel without functionalisation, which responded only to environmental changes. TWEEN 80 was used to functionalise both channels, creating a passivation layer that minimises the binding affinity with complex biofluid components. The sensor’s output is derived from the differential signals of both channels. This device could be mass-produced and used for early self-detection of cytokine levels in individuals with asymptomatic or mild COVID-19 through a dedicated Android app, which offered early warnings of condition deterioration. The biosensor’s design also enables its fabrication on flexible materials, making it suitable for wearable use in continuous cytokine monitoring across different stages of COVID-19, including moderate to critical cases, under various physical conditions [117].

##### Papillomavirus Detection

The human papillomavirus (HPV) is a common sexually transmitted virus that plays a critical role in the development of cervical cancer [118]. With over 100 identified types of HPVs, about half of them infect the genital area, and all share a genetic makeup of a circular double-stranded DNA genome that encodes approximately eight open reading frames (ORFs). Since only a tiny percentage of those infected display symptoms, molecular diagnostic techniques such as nucleic acid hybridisation assays and polymerase chain reaction (PCR) are used for early detection [119].

Research by Aspermair et al. [120] demonstrated the potential of using rrGrOfield-effect transistor (rGrO-FET) for HPV detection. The method involved using pyrene-modified rGrO, functionalised with an RNA aptamer Sc5-c3, to detect the HPV-16 E7 protein. The detection system operates by attaching an HPV-16 E7-specific aptamer to the rGrO-FET surface, which has been previously treated with a mixture of 1-pyrene butyric acid and pyrene-PEG at a 1:10 ratio via EDC/NHS coupling. This aptamer-functionalised rGrO-FET allows for real-time observation of the binding between the aptamer and the HPV-16 E7 protein with a detection threshold of around 100 pg$\;\cdot \;{\mathrm{mL}}^{-1}$ (1.75 nM) against a background of five blank noise signals, providing a 95% confidence level. The practicality of this approach for clinical use in point-of-care settings was assessed using saliva samples containing HPV-16 E7 protein, showing a linear detection range from 30 to 1000 nM. The aptamer-protein binding reaction’s dissociation constant ($K_{d}$) was 200 nM. The manuscript does not feature a dedicated future work section; nevertheless, the implications for subsequent research arise from the conclusions and discussions presented. Future directions may include the validation of the rGO-FET platform in larger clinical cohorts of saliva samples, extending beyond spiked specimens, to ascertain its diagnostic applicability. Additionally, further optimisation of sensor stability and reproducibility will be essential to underpin reliable long-term usage. The exploration of broader HPV screening scenarios within point-of-care diagnostics is also warranted.

Checkin et al. explored modified glassy carbon electrodes (GCEs) functionalised with porous rGrO (prGrO) and molybdenum sulphide (${\mathrm{MoS}}_{2}$) to selectively and sensitively detect HPV [121]. Specifically, the researchers covalently functionalised the electrode with an aptamer Sc5-c3, an RNA aptamer that targeted the human papillomavirus type 16 major capsid protein L1 (HPV-16 L1). By using differential pulse voltammetry (DPV) and an optimised sensor interface, a linear relationship was established between the concentration of HPV-16 L1 proteins (ranging from 0.2–2 ng$\;\cdot \;{\mathrm{mL}}^{-1}$ or 3.5 pM–35.3 pM) and the peak current density of a redox couple such as ferrocyanide ion (${\left[\mathrm{Fe}{\left(\mathrm{CN}\right)}_{6}\right]}^{4-}$) with the detection limit of 0.1 ng$\;\cdot \;{\mathrm{mL}}^{-1}$ (1.75 pM). The biosensor showed accuracy with recovery rates ranging from 97% to 105% on human saliva. Although there was a minor 5% decrease in function after the sensor was stored dry for a month, its stability remains uncompromised. The sensor was unaffected by interferents like ovalbumin (OVA) and human serum albumin (HSA), ensuring its selectivity in detecting HPV-16 L1. However, the inter-assay reproducibility indicated by RSD of 9.3% exhibited challenges in achieving consistent performance across different tests and hence could impact the accuracy of diagnoses. The authors suggest future studies to validate their aptamer-based prGO/MoS_2_ electrode platform across larger clinical samples to establish its practicality as an alternative to nucleic acid hybridisation assays, thereby advancing point-of-care diagnostics for HPV and related infections.

##### Influenza Virus Detection

To detect the influenza A (H1N1) virus, Joshi et al. developed an electrochemical immunosensor using a novel material: shellac-derived thermally reduced rGrO (TrGrO) [122]. The sensor achieved detection limits of 26 PFU/mL in phosphate-buffered saline (PBS) and 33 PFU/mL in diluted saliva, demonstrating good performance in both buffer and a complex medium. The device retained 90% of its initial signal after two weeks of storage. Moreover, the device exhibited selectivity, showing minimal response to other viruses like adenovirus, influenza B virus, and MS2 bacteriophage in electrochemical impedance spectroscopy (EIS) tests. The inter-assay variability is reduced, with an RSD value of 2.06%, indicating superior reproducibility compared to the HPV-16 L1 detecting sensor developed by Checkin et al. [121]. Joshi et al. [122] highlight the potential of shellac-derived thermally reduced graphene oxide for fabricating a wide variety of immunosensors, indicating its capability to extend detection to additional viral and bacterial biomarkers. They underscore the prospect of optimising biopolymer-based synthesis for cost-effective large-scale production.

##### Lysozyme Detection

Lysozyme, a vital antimicrobial enzyme produced by the immune system in response to attacks by pulmonary pathogens, has been the subject of numerous research studies highlighting its importance and prospective applications in medical diagnostics [114]. As an early example of a graphene-based lysozyme sensor, Xiao et al. (2013) developed an electrochemical platform by reducing graphene oxide (rGrO) on a glassy carbon electrode (GCE) [123]. The graphene surface was subsequently activated using EDC/NHS chemistry to covalently immobilise a lysozyme-binding aptamer (LBA). This aptasensor demonstrated high specificity for lysozyme in tests on human saliva and chicken egg white, achieving a linear detection range of 0.01 to 0.5 pM$\;\cdot \;L^{-1}$ and a signal-to-noise (SNR = 3)-based detection limit of 6 fM$\;\cdot \;L^{-1}$.

Building upon this foundation, Liu et al. further expanded the toolkit for lysozyme detection by introducing a biosensing strategy using a graphene oxide/ssDNA setup and a fluorescein-labelled anti-lysozyme DNA aptamer [124]. By employing GrO as a nano-quencher and the aptamer as a probe for target recognition and fluorescence signal transduction, this approach achieved unparalleled sensitivity among similar fluorometric “turn-on” techniques for lysozyme detection. The aptamer is specifically attached to lysozyme, causing it to detach from the GrO surface and activate the fluorescence signal. This method achieved a record-low detection limit of 21.8 pM with a detection range of 1.6 μL to 14.4 μL in saliva. Notably, the biosensor developed by Liu et al. presented a detection limit in the picomolar range, surpassing the sensitivity benchmarks established by previous methodologies, including that of Xiao et al. [123]. The effectiveness of the biosensor was validated through tests conducted on diluted saliva samples and standard buffer solutions, where it exhibited satisfactory recovery values and demonstrated exceptional specificity towards lysozyme. This finding was evidenced by an eight-fold increase in the relative fluorescence peak when compared against six control proteins, namely bovine serum albumin (BSA), interferon-gamma (IFN-γ), myoglobin (Mb), cytochrome C (Cyt C), and nuclear factor kappa-light-chain-enhancer of activated B cells (NF-κB), thereby underscoring the biosensor’s remarkable selectivity and potential utility in clinical diagnostics [124]. While the authors summarise the benefits of their methodology, an explicit future work paragraph is absent. However, inferred future directives can be drawn from the limitations discussed, suggesting a need for validation across a wider array of clinical samples (including saliva, serum, and urine). Furthermore, the investigation of this approach for alternative protein biomarkers beyond lysozyme is proposed, considering the generalisability of the aptamer/GO assembly platform. Additional optimisation of sensitivity and practical deployment for point-of-care diagnostics is also recommended.

##### Pseudomonas Aeruginosa Detection

Pseudomonas aeruginosa is a bacterium linked to potential chronic lung infections, secretes Pyoverdine (Pyo), a siderophore that chelates iron [125,126]. To take advantage of different nanomaterials’ combined properties, Cernat et al. (2018) developed a biosensing platform [127]. By depositing polypyrrole-3-carboxylic acid (Ppy-COOH) on graphene-modified screen-printed electrodes (SPEs) and decorating the platform with gold nanoparticles (Au NPs), they stabilised the composite structure and boosted the device’s sensitivity. The resulting biosensor is selective against ascorbic acid, acetylsalicylic acid, uric acid, NADH, dopamine, and glucose. It presented LOD in the micromolar range (0.33 μM) with a detection range between 1 and 100 μM. However, the device’s long-term stability was unsatisfactory. The stability test results demonstrated that the sensor maintained over 90% stability of the initial signal following the first four trials, with a decrease of up to 30% observed by the eighth test. To assess reusability, the sensor underwent weekly testing over two weeks. The recorded signal increased 43.42% after one week and 61.12% after two weeks. Furthermore, the sensor was evaluated using human saliva, and commercial serum spiked with 25 μM Pyo, following dilution with 0.02 M PBS at pH 7.4. The average recovery rates were 105% for the saliva sample and 100.47% for the serum sample spiked with Pyo. To address the limitation regarding long-term stability, the same group developed another version of the sensor based on the same nanocomposite platform [128]. The new sensor showed improved recovery, with 102.12% retention of its initial performance, and substantially increased long-term stability, retaining 91.43% of its initial performance after 30 days in dry storage. It was reported that under optimal conditions, the electrochemical signal obtained due to the oxidation of Pyoverdine is proportional to the concentration with a linear spectrum ranging from 1 to 100 $\mu M\;\cdot \;L^{-1}$, and this relationship was characterised by a detection threshold of 0.33 $\mu M\;\cdot \;L^{-1}$, indicating the sensitivity of the method. Nevertheless, the device exhibited poor intra-assay reproducibility after the fifth consecutive test, indicating limitations of the nanocomposite platform in regeneration and successive uses. However, the inter-assay RSD of 5.6% falls within typical values for biosensors. The authors state that their innovative technique holds promise for future applications in medical and environmental contexts. They emphasise that the outstanding reproducibility and high storage stability of the nanocomposite platform render it suitable for diverse analytical applications.

##### Drug Detection

There has been a growing interest in using saliva as a bodily fluid for quickly screening drugs and hazardous substances and therapeutic drug monitoring [129], and considerable developments in designing wearable and portable biosensors to detect these substances. However, only a few research groups have concentrated on creating graphene-based devices.

Parate et al. [130] developed an electrochemical biosensor to measure exposure to smoke and tobacco derivatives. The biosensor specifically targets biomarkers in smokers, such as cotinine, various tobacco alkaloids, and nicotine metabolites. The device utilised Screen-printed Carbon Electrodes (SPCEs) enhanced with graphene and platinum nanoparticles (PtNPs) and biofunctionalised with an electrodeposited molecularly imprinted polymer (MIP). With a LOD of 0.33 nM, the biosensor operated across a linear range of 1–100 nM and exhibited a sensitivity of 1.89 $\mu A\;\cdot \;{\mathrm{decade}}^{-1}$ in actual saliva samples. Selectivity was efficiently tested against similar compounds, such as nicotine and myosmine.

The analysis of neurological drugs is crucial in the treatment of major neurological disorders like Alzheimer’s and Parkinson’s conditions [131], as well as in migraine, status epilepticus, stroke, and traumatic brain injury [132], as drug dosage significantly impacts body fluids’ dynamics. Rajendran et al. used a bottom-up approach to develop a 2D hybrid film that combined MXene and graphene (MX/Gr), which was then utilised in an electrochemical transducer to detect nicotine electrochemically [131]. The presence of graphene improved the stability of the MXene dispersion through its surface functional groups. Furthermore, they developed a GCE modified with the MX/Gr hybrid film composite for selective nicotine detection in a phosphate buffer solution (0.1 M PBS, pH approximately 7.4). The MX/Gr/GCE sensor showed a linear response to nicotine concentrations ranging from 1 μM to 55 μM and 30 nM to 600 nM, with LOD being 290 nM and 0.28 nM, respectively, as measured by differential pulse voltammetry (DPV) and amperometry under optimised conditions.

Fu et al. developed a graphene-based biosensor for detecting ketamine in saliva samples [133]. Their research described using a molecularly imprinted electrochemical sensor, which achieves remarkable sensitivity and selectivity in identifying ketamine. The core of this sensor’s functionality lies in its biomimetic recognition layer, which is ingeniously fabricated through the UV-triggered polymerisation of methacrylic acid (MAA) and ethylene glycol dimethacrylate (EGDMA). This process takes place atop a modified screen-printed electrode, itself incorporated with a metal–organic framework/graphene nanocomposite (MOFs@Gr), resulting in a synergistic enhancement of the sensor’s stability and adherence properties for the imprinted membranes. The sensor presented a detection threshold of $4\times 10^{-11}\;M\;L^{-1}$, spanning a dynamic range from $1\times 10^{-10}\;M\;L^{-1}$ to $4\times 10^{-5}\;M\;L^{-1}$, presenting its sensitivity, selectivity, and stability. The authors indicate that their sensor exhibits substantial prospects for the detection of real human serum and saliva samples. In their conclusions, they suggest that this sensor could serve as a promising method for the future detection of trace ketamine in urine and saliva samples.

In 2017, Mohamed et al. developed an electrochemical sensing system to simultaneously measure benzocaine (BEN) and antipyrine (ANT) [134]. The system employs a carbon paste electrode (CPE) enhanced with titanium dioxide (${\mathrm{TiO}}_{2}$) nanoparticles and GrO nanosheets, referred to as ${\mathrm{TiO}}_{2}$-GrO/CPE. This sensing system presented a linear detection range and LOD of $1.0\times 10^{-6}$ to $1.0\times 10^{-4}$ M and $2.48\times 10^{-7}$ M, respectively, for BEN, and $1.2\times 10^{-8}$ to $8.0\times 10^{-5}$ M and $3.00\times 10^{-9}$ M, respectively, for ANT. The platform had a linear response across three orders of magnitude and demonstrated recovery rates of 99.62%, with stability ranging between 98.8 and 99.1% over 30 days. Intra-assay RSDs were 1.9% for BEN and 2.2% for ANT, with inter-assay RSDs of 2.5% for BEN and 1.8% for ANT, confirming the platform’s precision in detecting each drug. The platform’s selectivity was also tested, showing effective detection of one analyte in the presence of various interfering substances. The authors conclude by indicating that their fabricated sensor possesses potential for future applications. They propose its utilisation as a clinical assay and for quality assurance (QA) in pharmaceutical products.

##### Cardiovascular Diseases Detection

Cardiovascular diseases are among the leading causes of death. The earlier these diseases can be identified, the better it is for lowering mortality rates through successful and appropriate treatments. Cardiac troponin I (cTnI) is a crucial protein in the heart that is a precise biomarker for detecting acute myocardial infarction and resulting damage to cardiac muscle [135].

Chekin et al. contributed to this field by demonstrating the usefulness of nitrogen-doped reduced rGrO(N-prGrO) in detecting and measuring cardiac troponin I (cTnI) in conditions that replicate those found in the human saliva [136]. By non-covalently attaching 1-pyrene carboxylic acid (py-COOH) and poly(ethylene glycol) modified pyrene (py-PEG) to N-prGO, they were able to attach the highly selective Tro4 aptamer for cTnI covalently. This method enabled the development of a label-free electrochemical sensor that employs differential pulse voltammetry (DPV) to detect cTnI. Concentrations of cTnI lower than $1\;\mathrm{pg}\;\cdot \;{\mathrm{mL}}^{-1}$ for a healthy patient and $675\;\mathrm{pg}\;\cdot \;{\mathrm{mL}}^{-1}$ for patients diagnosed with acute myocardial infarction (AMI) were measured in human saliva. The sensor remained stable for one month with minimal performance degradation of only 8%. Its sensitivity, determined by the slope of the current-concentration calibration curve, is $41\;\mu A\;\cdot \;{\mathrm{cm}}^{-2}$ per decade. It selectively detected cTnI even with potential interfering substances like BNP, BSA, and lysozyme. The authors opine that their sensor demonstrates applicability for cTnI detection in both human blood and saliva samples. They propose that the sensor may provide excellent prospects for sensitive screening and routine monitoring of cTnI in clinical settings. Additionally, they highlight the appeal of a painless and non-invasive saliva sensing analysis, particularly for elderly patients.

Yoo et al. developed a method for increasing graphene electrode-based EIS biosensors’ sensitivity and electroactive surface area without relying on external structuring techniques such as patterning or pore generation [137]. The researchers utilised GrO nanosheets, which they functionalised with octadecyl amine (ODA) groups to adjust the inter-sheet spacing. These nanosheets were then put together into stacked films, which were thermally reduced to create a roughened surface that enhances the electroactive surface area and sensing sensitivity. Using electrochemical impedance spectroscopy (EIS) measurements, they tested the sensing capabilities of rGO electrodes with varying inter-sheet distances to detect myoglobin (Mb), a biomarker of acute myocardial infarction, as proof of concept. The stacked GrO nanosheets that underwent controlled interlayer alkylation proved a successful strategy for generating rGrO electrodes with a significantly enhanced and/or controlled surface area, resulting in a sensing performance with a detection range and limit of 5 pM–10 nM and 2.37 pM concentration of Mb, respectively, and demonstrating satisfactory recovery rates (91–111%) in actual saliva samples. The researchers state that their proposed strategy is broadly applicable to a variety of target molecules and is anticipated to establish a general platform for biomedical and environmental sensors. They further suggest that this platform holds significant promise for the early diagnosis of acute myocardial infarction. Additionally, the authors assert that their strategy could be seamlessly integrated into a diverse range of next-generation, high-performance biomedical and environmental sensor applications.

##### Cortisol Detection

Cortisol plays a vital role in the response to psychological stress. Scientific research has shown that cortisol levels in saliva accurately reflect serum levels. This discovery encourages researchers to develop biosensors that detect cortisol levels non-invasively through saliva analysis [114]. Zhang et al. developed a portable test that accurately measures salivary cortisol levels using a liquid gate GFET (LG-GFET) as a sensitive material [138]. The LG-GFET was brought into existence through the utilisation of advanced printing techniques. They achieved this by modifying liquid exfoliation techniques to create graphene ink, which was then printed using direct-ink-write technology to produce the LG-GFETs. These sensors were further improved and functionalised with tetrakis(4-carboxyphenyl) porphyrin and a cortisol aptamer before assessing their sensitivity, selectivity, and durability performance. From the result, it was found that the sensor presented a linear sensitivity range of 0.08–800 nM and was suitable for monitoring daily cortisol levels. The efficiency of the sensor was tested against different backgrounds, such as PBS, artificial saliva, and natural saliva, demonstrating its sensitivity, selectivity towards cortisol, and resilience against pH variations. In addition, the sensor’s ability to monitor circadian rhythms was conceptually proven through its application. Zhang and Jia propose that their platform offers an alternative solution for the development of analogous household medical devices. They assert that their work may pave the way for the creation of Lg-GFET-type products tailored for use in various salivary biomarker assays.

In their investigation, Zubarev et al. studied the sensing potential of a composite fabricated from multi-layer graphene intercalated with pyrrole to devise a cost-efficient, susceptible material for cortisol detection [139]. The synthesis involved the dispersion of graphene nanoplatelets into pyrrole within a 1 M nitric acid (${\mathrm{HNO}}_{3}$) solution, followed by sonication using an intense ultrasound probe for 10 min. Subsequently, the mixture underwent centrifugation at a rate of 4000 revolutions per minute for 30 min. CV was employed to initiate polymerisation. The graphene-pyrrole composite that emerged, as a result, demonstrated sensitivity towards detecting subnormal levels of cortisol in synthetic saliva, which closely replicated the concentrations found in human saliva specimens. Raman spectroscopy and modelling the interaction between the composite’s sensitive layer and cortisol using MarvinBeans software demonstrated its efficient sensitivity. Specifically, it could detect cortisol concentrations as low as 0.5 ng$\;\cdot \;{\mathrm{mL}}^{-1}$. The authors explicitly discuss the forthcoming steps in the development of their sensor, which encompass conducting interferent measurements with other molecular constituents present in human saliva, establishing the reproducibility of the synthesis method through testing a greater number of sensors, and evaluating the sensor’s stability under varying conditions. A final stated objective includes the miniaturisation of the sensor for integration into a wearable device.

Khan et al. developed a paper-based biosensor that is affordable, fast, and efficient for real-time and POC applications to detect cortisol [140]. The sensor’s electrode is coated with poly(styrene)-block-poly(acrylic acid) (PS67-b-PAA27) and graphene nanoplatelets (GNPs) onto filter paper, which enhances the sensitivity of the immune response. The sensor was also integrated with a portable electronic printed circuit board (PCB) that eliminates the need for additional electrolyte solution once the saliva sample is applied to the sensor. The biosensor chip had a detection capability ranging from 3 pg$\;\cdot \;{\mathrm{mL}}^{-1}$ to 10 μg$\;\cdot \;{\mathrm{mL}}^{-1}$, with a minimum detection limit of 3 pg$\;\cdot \;{\mathrm{mL}}^{-1}$ and a sensitivity of 50 $\Omega \;{\left(\mathrm{pg}/{\mathrm{mL}}^{-1}\right)}^{-1}$. They compared the results from the biosensor chip to the enzyme-linked immunosorbent assay (ELISA) method and found a variance of only 1.5–3.7%, indicating high accuracy. This achievement was made possible by attaching an anti-cortisol antibody (anti-CAB) to gold (Au) microelectrodes using 3,$3^{\prime }$-dithiodipropionic acid di(N-hydroxysuccinimide ester) (DTSP) as a coupling agent to create a self-assembling monolayer (SAM). The comparison of this cortisol sensor with the traditional ELISA method yielded a regression value of 0.9951, confirming its effectiveness. In a subsequent study, Khan et al. [141] introduced another biosensor, maintaining graphene as the core sensing element to analyse cortisol levels in human saliva for POC testing and on-site recording. The chip was connected to a high-precision impedance converter system, and electrochemical impedance spectroscopy was performed to assess cortisol levels in seven saliva samples. The chip was optimised with a sensitivity enhancer, bovine serum albumin, and two other parameters: anti-cortisol antibody covalently attached to micro-Au electrodes and saliva sample incubation time to improve cortisol recognition and achieve greater accuracy. The sensor presented a lower detection limit of 0.87 ± 0.12 pg$\;\cdot \;{\mathrm{mL}}^{-1}$, with a wide range from 1 pg$\;\cdot \;{\mathrm{mL}}^{-1}$ to 10 ng$\;\cdot \;{\mathrm{mL}}^{-1}$ and an accuracy of 96.3% compared to the gold standard ELISA test. Previous biosensors also reported similar results. The scientists declare that the proposed device is relevant for point-of-care testing (POCT) aimed at in vitro psychobiological investigations concerning patient cortisol levels in saliva. They surmise that the device holds great potential for further testing and implementation across multiple clinical sites. As a long-term aspiration, the authors envision the development of an implantable sensor chip in the future.

Simultaneously, Kim et al. [142] proposed developing a cortisol-sensing device based on a chemo-resistive detection mechanism. The sensor utilised a cortisol monoclonal antibody (c-Mab) covalently bonded to rGrO channel, which served as the electrical detection component. The change in resistance observed in the rGrO channel-based chemiresistor sensor determined the specific interaction between the c-Mab and cortisol. The detection threshold was obtained as low as 10 pg$\;\cdot \;{\mathrm{mL}}^{-1}$ (27.6 pM) with a detection range of 1 to 10 ng$\;\cdot \;{\mathrm{mL}}^{-1}$. The researchers also measured cortisol levels in human saliva samples and buffer solutions from rat adrenal gland slices under acute conditions. These slices secreted cortisol in response to adrenocorticotropic hormone (ACTH) through the chemiresistor, indicating precise cortisol detection. Even among various neuroendocrine substances, the rGrO chemiresistor effectively identified cortisol concentrations. Although the study did not specify a correlation coefficient or accuracy figure, the biosensor was validated against the ELISA gold standard and presented stability over several months. Its selectivity was affirmed in a neuro-cell culture matrix containing various neuroendocrine substances, neurotransmitters, and proteins. The authors articulate that their study aims to introduce a new protocol for cortisol sensor development. They specify that this protocol will apply to point-of-care testing (POCT) focused on salivary cortisol, in vitro psychobiological studies concerning cortisol induction, and the future development of an implantable sensor chip.

Furthermore, Kim et al. [143] designed an electrochemical biosensor to detect cortisol. This biosensor was built with an electrochemical sensing mechanism that combines an rGrO electrode, a dual-purpose protein interlayer, and a specific antibody probe that precisely targets cortisol molecules. The interlayer, made of thermally denatured bovine serum albumin (d-BSA), was applied directly to the rGrO electrode’s surface through π-stacking interactions. This setup created covalent sites for attaching anti-cortisol antibody probes and prevents the non-specific adsorption of other substances. The biosensor structure (antibody/d-BSA/rGO) detected cortisol with picomolar sensitivity, and it had a linear dynamic range from 10 pM to 100 nM with a LOD of 10 pM, as per the Electrochemical Impedance Spectroscopy (EIS) analysis. The biosensor was highly selective for cortisol over similar molecules, such as aldosterone and progesterone, with minimal cross-reactivity. It was reported that the sensor could recover cortisol levels in human saliva samples, even in the presence of various interfering substances. Without gold standard validation, the sensor underwent testing for recovery in human saliva samples, yielding recovery rates within the 92–103% range, marking the highest recovery rates reported among the discussed cortisol sensors. Notably, it was found that when cortisol monitoring occurred in the presence of aldosterone and progesterone, the sensor’s resistance increment in response to cortisol detection was eightfold, presenting selectivity for the target analyte. The researchers assert that their demonstrated strategy is poised to enhance the efficient interfacing of diverse biomolecular probes with graphene-based and other electrode materials possessing hydrophobic surfaces. They conclude that their sensor shows considerable potential for practical applications where determining cortisol levels is critical for diagnosing stress-related disorders.

##### Serotonin Detection

Serotonin is a neurotransmitter present in the central nervous system and peripheral tissues and influences behaviour and mood, the learning process, circadian rhythms, and anxiety levels. Adumitrăchioaie et al. developed an immunosensor that utilised graphene oxide-chitosan on gold screen-printed electrodes to detect serotonin [144]. The sensor demonstrated a 0.05 μA·$\mu M^{-1}$ sensitivity that enabled it to detect substances from 10 nM to 100 mM and also features a low detection limit of 3.2 nM. Even after one month of storage and exposure to potentially interfering substances, it retained more than 91% of its effectiveness, further underlining its potential impact on health monitoring. This research serves as a foundational basis for the development of point-of-care devices aimed at the rapid, selective, and cost-effective detection of serotonin. Subsequent investigations should centre on enhancing the analytical performance and long-term stability of the sensor. Such advancements would position the platform more favourably for practical applications in clinical and diagnostic environments.

##### L-Tryptophan Detection

L-tryptophan aids in serotonin production and, apart from cortisol and serotonin, is also considered a marker for mental health issues such as mood fluctuations and depression [114]. Nazarpour et al. developed a method for directly measuring L-tryptophan (Try) by utilising *Eucalyptus tereticornis* leaves as a green reducing agent to create reduced graphene oxide/gold nanoparticles (rGrO/AuNPs) [145]. The developed nanocomposite was utilised as an electroactive and sensitive layer on the surface of screen-printed electrodes for the electrochemical oxidation detection of Try in biological samples. Differential pulse voltammetry (DPV) and CV provided a reliable analytical signal for Tryptophan’s oxidation at 0.65 V. The detection sensitivity was enhanced through a central composite design utilising response surface methodology (RSM). The method showed a linear response for Try concentrations ranging from 0.5 to 500 μM·$L^{-1}$ (with a correlation coefficient, R, of 0.9976) and achieved an LOD and LOQ of 0.39 and 1.32 μM·$L^{-1}$, respectively, with recovery rates ranging from 95 to 108%. Optimal conditions were achieved by utilising a Britton Robinson buffer at pH 6.0, a ratio of AuNPs to rGrO (*W*/*W*) of 4, and a nanocomposite volume of 5 μL dropped onto the electrode. The sensor’s selectivity was confirmed against a range of substances without significant interference, ensuring accurate detection of L-tryptophan among common compounds found in saliva. Although the manuscript lacks a distinctly labelled “Future Work” section, potential directions are inferred from the conclusion and discussion. Future research endeavours could investigate more sustainable synthesis methods for nanocomposites employing *E. tereticornis* and expand the sensor’s applicability to other biomarkers and clinical contexts. Further optimisation of sensor parameters and adaptation to portable diagnostic devices are also advised.

##### Glucose Detection

Cardoso et al. demonstrated the development of biosensors for analysing biological fluids using 3D printing technology [146]. They utilised fused deposition modelling to create sensing platforms from polylactic acid filaments infused with graphene (Gr-PLA). A glucose biosensor was engineered on the Gr-PLA surface using chronoamperometry and was employed to detect glucose in blood plasma. The polymer matrix’s oxygenated groups improved the biosensor’s performance by enabling enzyme immobilisation through crosslinking with glutaraldehyde. This biosensor exhibited LOD of 15 μM·$L^{-1}$, demonstrated inter-day and intra-day precision below 5%, and had recovery rates ranging from 90 to 105% in plasma analysis. The paper concludes by presenting future perspectives for this work, particularly the development of wearable biosensors, owing to the flexible, biodegradable, and biocompatible nature of their 3D-printed G-PLA sensors. They assert that this technology has the potential to facilitate the production of (bio)sensors on a large scale and in various dimensions.

Nugba et al. [147] developed a nonenzymatic glucose detection electrode using a rapid galvanic pulse electrodeposition method. The technique involved creating finely dispersed copper nanoparticles on the surface of a three-dimensional laser-induced graphene (LIGr) structure. This process uses a ${\mathrm{CO}}_{2}$ laser to generate graphene fibres on a flexible polyimide base, forming a conductive LIGr electrode platform for depositing the copper nanoparticles (CuNPs). The study emphasised how laser-engraved porous graphene (LIGr) on a polyimide base, followed by pulse current deposition (PCD) of CuNPs smaller than 29 nm, was effective for nonenzymatic glucose sensing. The resulting electrode increased glucose detection capabilities with a sensitivity of 2665 μA$\;\cdot \;{\mathrm{mM}}^{-1}$$\;\cdot \;{\mathrm{cm}}^{-2}$, a response time of under 5 s, a detection range of 0.03 mm–4.5 mm, and a detection limit of 0.023 μM. Moreover, the electrode demonstrated selective glucose detection in the presence of substances such as ascorbic acid and urea, enabling it to sense glucose in saliva with a 97% accuracy rate. The authors conclude their study by underscoring the future applicability of their sensor for practical use. They assert that the electrode exhibits significant promise for sensing low glucose concentrations in saliva, positioning it as suitable for real-world diagnostic applications. Additionally, they highlight the sensor’s robust capability to accurately detect and measure glucose in biological samples.

Jia et al. developed a technique for directly quantifying glucose using microfluidic paper-based analytical devices (μPADs) coated with GrO and smartphone-based colourimetric detection [148]. The GrO deposition improved the efficiency and homogeneity of colour distribution of the reagents, enhancing the performance of the glucose assay. A self-developed app was used to quantify glucose concentrations within the physiological range automatically. The modification with GrO eliminated potential enzyme cross-contamination without requiring a linker, binder, or retention aid. The system was calibrated with standard glucose buffer solutions, and its limit of detection and linear dynamic range were determined to be 0.02 mM and 0∼1 mM, respectively, which makes it suitable for analysing glucose concentrations in the clinically relevant range. The system was also tested with artificial saliva, and the results obtained were in reasonable agreement with the actual concentrations, demonstrating its portability and sensitivity for quantifying glucose concentrations. Jia et al. indicate that their approach could be utilised for a wide array of other bioanalytical applications. Moreover, they suggest that the advancements achieved in their study have the potential to facilitate non-invasive glucose point-of-care diagnostics.

Gao et al. developed a noninvasive glucose sensor that utilises Copper(I) oxide (${\mathrm{Cu}}_{2}$O) nanocubes and a flexible graphene substrate [149]. By using an electrochemical method, the ${\mathrm{Cu}}_{2}$O nanocubes were deposited onto the graphene strip substrate to produce a near-homogeneous monolayer of ${\mathrm{Cu}}_{2}$O-shell Cu-core nanocubes that are approximately 50 nm in size. The optimal electrodeposition conditions of −1.0 V vs. Ag/AgCl, 1 mM [${\mathrm{Cu}}^{2+}$], and 100 s deposition time at room temperature were utilised to achieve this result. The ${\mathrm{Cu}}_{2}$O nanocubes/Gr system was a sensor with a detection range of 0.002–17.1 mM, with LOD and sensitivity of 0.23 μM and 36.4 μA$\;\cdot \;{\mathrm{mM}}^{-1}$$\;\cdot \;{\mathrm{cm}}^{-2}$, respectively, making it ideal for saliva-range glucose sensing. In comparison to other nonenzymatic sensors, including bare graphene, graphene sputter-coated with a Cu film, and conventional enzymatic sensors such as glucose oxidase immobilised on graphene (with and without Nafion), this nonenzymatic glucose sensor performed considerably better. Moreover, this sensor was tested on a saliva sample and achieved 95% accuracy. The researchers assert that their sensor demonstrates high sensitivity, appropriate for detecting glucose within the saliva range, and has been favourably tested using authentic saliva samples. They propose that the Cu_2_O nanocubes/graphene glucose sensor provides a sensitive, non-invasive alternative for monitoring saliva-range glucose levels, with minimal interference for patients with diabetes.

In 2024, Shang et al. introduced a 3D electrode composed of rGrO infused with MXene (${\mathrm{Ti}}_{3}C_{2}$) and AuNPs for non-invasive glucose measurement in saliva [150]. The Au/rGrO-${\mathrm{Ti}}_{3}C_{2}$ electrode exhibited exceptional performance in detecting glucose levels ranging from 10 μM to 21 mM at an operational voltage of 0.6 V. It achieved a glucose detection threshold of 3.1 μM and a sensitivity rate of 355 μA$\;\cdot \;{\mathrm{mM}}^{-1}$$\;\cdot \;{\mathrm{cm}}^{-2}$. With its ability to detect glucose in both artificial and actual saliva samples, the sensor is sensitive to concentrations as low as 10 μM. These results indicate that the Au/rGrO-${\mathrm{Ti}}_{3}C_{2}$ electrode is a promising candidate for saliva-based glucose monitoring. This non-invasive approach could significantly improve diabetes management, offering a painless alternative to traditional blood glucose monitoring. The manuscript contends that the electrode developed in their research holds significant potential for enhancing diabetes monitoring efficiency through non-invasive glucose detection in saliva. They conclude that their work could offer a novel approach for non-invasive glucose detection, thereby expediting the diagnosis of diabetes.

##### Nitrite and Uric Acid(UA) Detection

**Figure 6:**
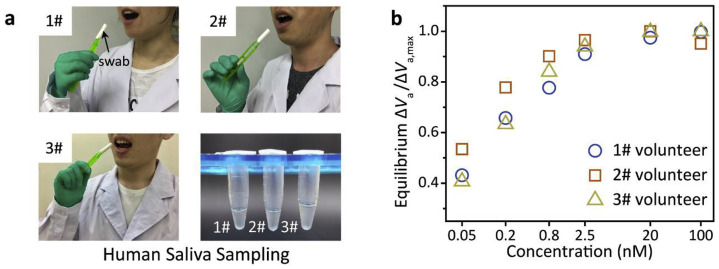
(**a**) Illustration of saliva collection from three volunteers, with centrifuged suspensions shown after sampling. (**b**) Normalised variation of the electrochemical response ($\Delta V_{a}/\Delta V_{a,\max}$) across a concentration gradient of IL-6 (50 pM to 100 nM) in the collected saliva samples. (Reproduced from [152] with permission from Elsevier).

**Figure 7:**
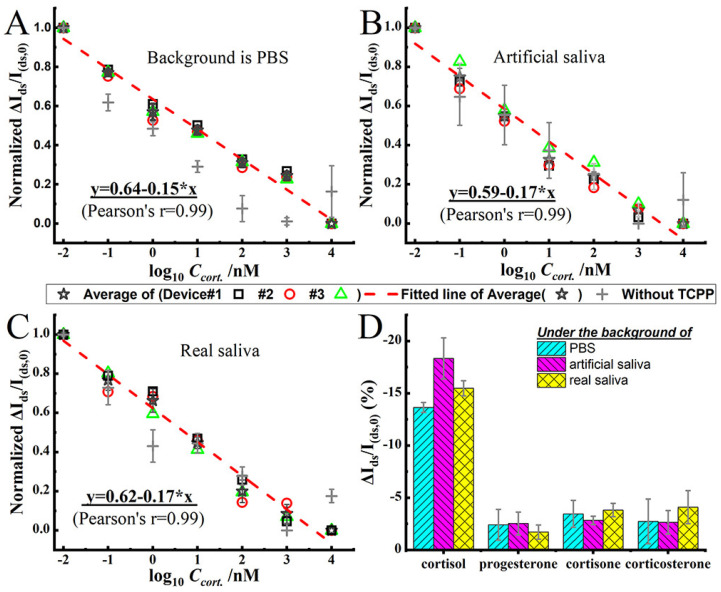
Performance of LG-GFET cortisol biosensors under distinct matrices: (**A**) PBS, (**B**) synthetic saliva, and (**C**) authentic human saliva, across cortisol concentrations ( $10^{-2}$ to $10^{4}$ nM). (**D**) Specificity assessment against structurally related steroids (cortisone, corticosterone, progesterone; 10 nM) in each matrix. Responses quantified via normalised drain current shift ($\Delta I_{\mathrm{ds}}/I_{\mathrm{ds},0}$), where $I_{ds,0}$ denotes baseline current at zero cortisol. Error bars indicate standard deviation (n = 3). (Reproduced from [138] with permission from ACS Publications).

**Figure 8:**
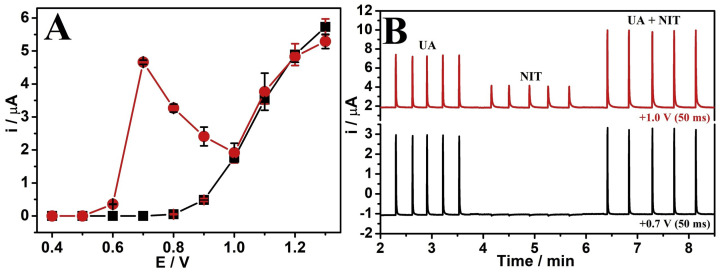
(**A**) Hydrodynamic voltammograms plotting peak current against applied potential pulses (70 ms duration) for UA (•) and NIT (◼) in solution (10 μM$\;\cdot \;L^{-1}$). (**B**) Amperometric traces (n = 5) for solutions containing UA (20 μM$\;\cdot \;L^{-1}$), NIT (20 μM$\;\cdot \;L^{-1}$), or their equimolar mixture. Conditions: BR buffer (pH 2), flow rate 280 μL $s^{-1}$, injection volume 100 μL. (Reproduced from [146] with permission from ScienceDirect).

**Figure 9:**
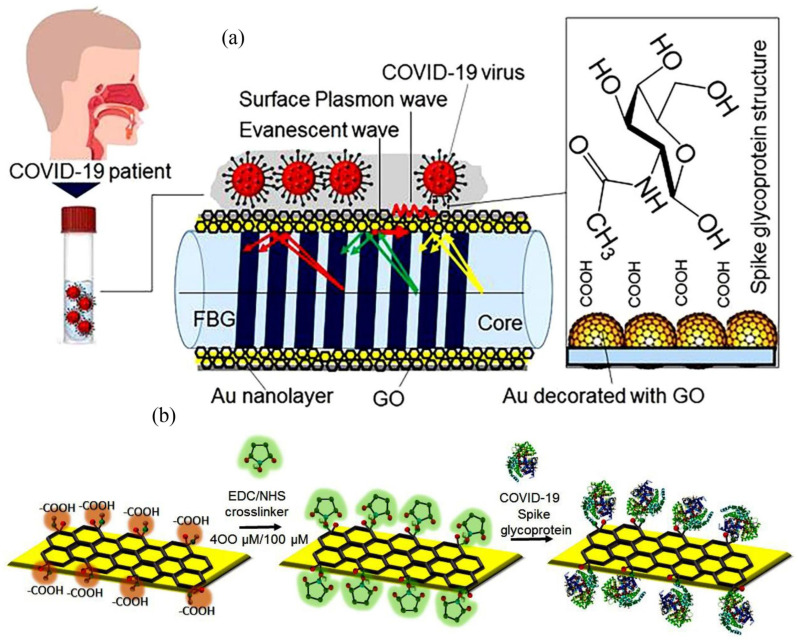
(**a**) Schematic mechanism illustrating binding of the SARS-CoV-2 spike glycoprotein with activated GO prepared using EDC/NHS chemistry. (**b**) Overall illustration of the sensing concept.(Reproduced from [115] with permission from ScienceDirect).

**Figure 10:**
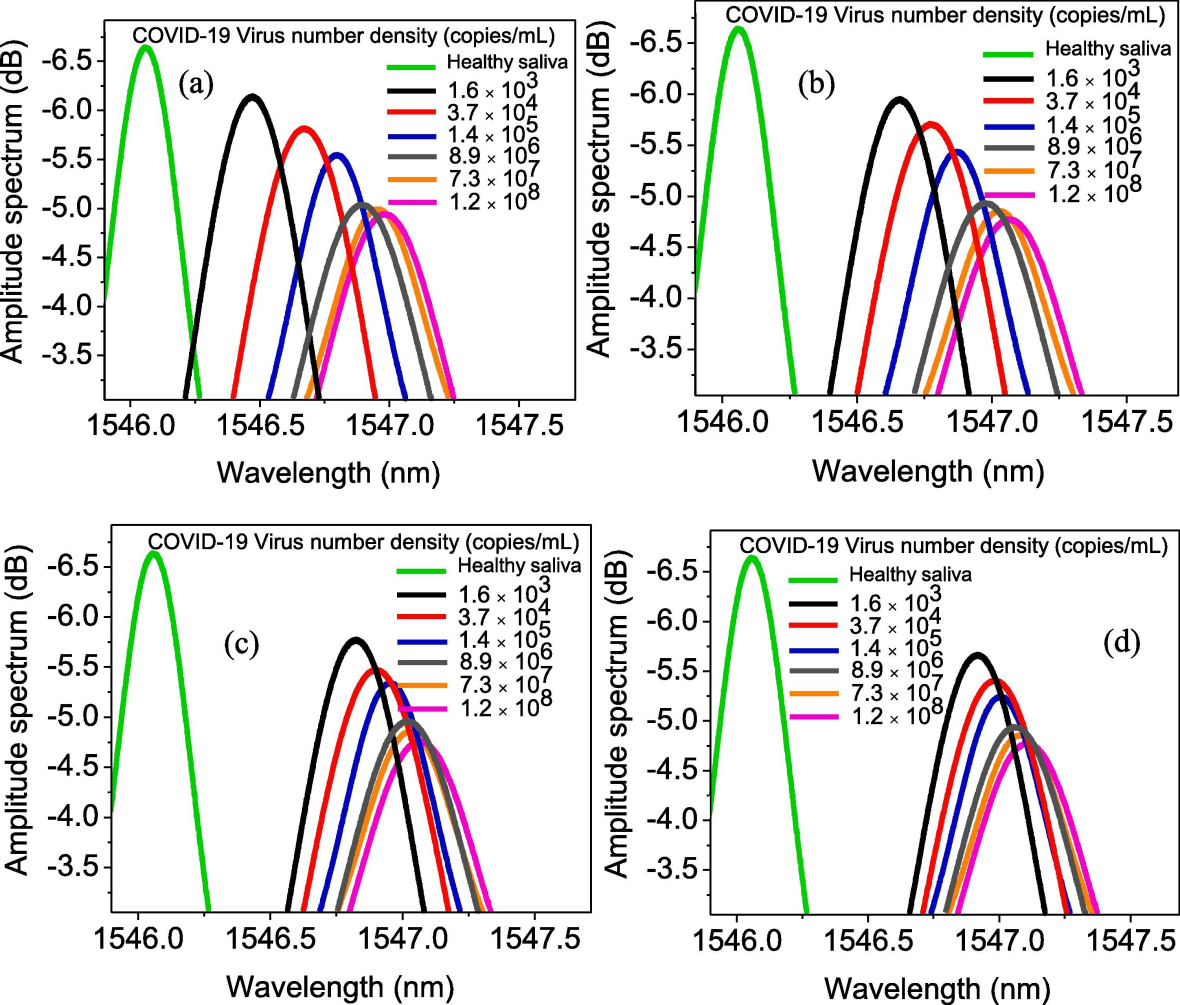
Spectral response of GO-decorated Au/FBG probes immersed in varying COVID-19 virus densities after (**a**) 10 s, (**b**) 20 s, (**c**) 50 s, and (**d**) 100 s. (Reproduced from [115] with permission from ScienceDirect).

The biosensor developed by Muñoz et al., along with glucose, also demonstrated the ability to detect nitrite and uric acid (UA) [146]. The mechanical polishing and solvent immersion as surface treatments improved the 3D-printed sensor’s electrochemical properties for directly detecting nitrite and uric acid. They utilised differential-pulse voltammetry and multiple-pulse amperometry under flow conditions to detect these substances in saliva and urine, providing amperometric detection in a linear range of 0.5–250 μM$\;\cdot \;L^{-1}$ for both analytes, LODs of 0.02 μM$\;\cdot \;L^{-1}$ for UA and 0.03 μM$\;\cdot \;L^{-1}$ for nitrite, and exhibiting high precision with an RSD less than 2.1%. Figure 6, Figure 7, Figure 8, Figure 9 and Figure 10 present different graphene-based non-invasive saliva sensors for detecting viruses and biomolecules.

In the study conducted by Huang et al., an approach to fabricating paper-based sensors was explored through the electropolymerisation of a composite material comprising poly (3,4-ethylene dioxythiophene) (PEDOT) and GrO onto an indium tin oxide (ITO) substrate [151]. The primary objective of this innovation was to develop a reliable method for quantifying UA concentrations in authentic human saliva specimens. The methodology adopted ensures a homogeneous distribution of the nanocomposite layer on the electrode surface, an aspect critically evaluated through its application in detecting UA in synthetic saliva. The sensor exhibited exceptional electrochemical sensitivity toward UA, alongside notable specificity and durability under test conditions. This paper-based analytical device was subsequently applied to the analysis of UA in unprocessed, undiluted human saliva samples, where it demonstrated electrocatalytic efficiency, reproducibility, and stability over time. The detection capabilities of this sensor span a concentration range of 2 to 1000 μM, with a lower detection limit of 0.75 μM, encompassing the typical UA concentration range present in human saliva. The text mentions that the developed device shows promise for the non-invasive monitoring of salivary uric acid in humans. Furthermore, they conclude that it represents a valuable method for non-invasive evaluation of salivary uric acid in clinical diagnostics.

##### Carbonic Anhydrase 1 Detection

The potential of human carbonic anhydrase 1 (CA1) as a biomarker for various diseases, including cancers, pancreatitis, diabetes, and Sjogren’s syndrome, has been suggested [153]. However, the widespread use of this marker in practical clinical applications has been hindered by the lack of an inexpensive, fast, precise, and user-friendly method for measuring CA1. Kumar et al. developed an electronic biosensor using the GFET combined with specific RNA aptamers to detect the CA1 enzyme without labels. A liquid gating method was utilised for detection to preserve the natural structure and functionality of the aptamers and CA1 while ensuring minimal operational voltage. The biosensor measured the change in GFET’s Dirac voltage (VD), effectively indicating the charge variations due to CA1 binding without being affected by resistance-increasing disorders. The study also examined the impact of two different concentrations of phosphate buffer saline (PBS) on the device’s sensitivity. The interaction between CA1 and the aptamers resulted in a positive shift in the VD, which varied according to the target concentration. The aptamer-based GFET biosensors demonstrated a high affinity for binding (KD) of approximately 2.3 ng$\;\cdot \;{\mathrm{mL}}^{-1}$ (70 PM), with a sensitivity of 65.4 mV/decade, and were specifically responsive to the CA1 enzyme, a biomarker associated with multiple diseases. The developed GFET biosensor identified the presence of CA1 within 30 min in PBS and 60 min in saliva, requiring only 40 μL of the sample [153]. Further investigations are necessary to ensure minimal sensitivity to other enzymes and bioactive molecules. The demonstrated G-FET biosensor for CA1 in saliva highlights the potential to extend this methodology towards the detection of other carbonic anhydrases and related biomarkers through the design of specific RNA aptamers.

##### Tumour Detection

The release of interleukins, a cytokine produced by the immune system, has been recognised as an important factor in cancer progression [114]. Kumari et al. (2016) observed that Interleukin-6 (IL-6) is consistently found in tumours, highlighting its potential as a key biomarker for cancer diagnosis and monitoring [154]. Interleukin-8 (IL-8) has also been linked to tumour progression in various cancers, including oral cancer [114]. In 2019, Hao et al. introduced an online method for identifying cytokine biomarkers in saliva [152]. This approach employed an aptameric GFET with a buried-gate geometry and ${\mathrm{HfO}}_{2}$ as the dielectric layer. Furthermore, online signal processing circuits were utilised to transduce and process signals that reflect cytokine concentrations. It was reported that the signal could be wirelessly transmitted to a smartphone or cloud server via Wi-Fi to visualise the cytokine concentration change trend. The system’s sensing capability was evaluated using IL-6 as a representative. The experimental findings showed that the nanosensing system reacted to changes in IL-6 concentration within 400 s from a 30 μL saliva sample, with a detection limit as low as 12.2 pM and a detection range between 0.05 and 0.84 nM. The device’s selectivity was demonstrated against similar molecules like EGF and GH, with minor voltage changes compared to IL-6. The observed response signal exhibited fluctuations of less than 7.47%, likely attributable to interferences stemming from the heterogeneity of real saliva samples. Future efforts will focus on enhancing sensing stability by safeguarding the graphene surface from interactions with background non-target molecules.

##### Cancer Detection

This section focuses on the detection of various types of cancer.

Lung Cancer Detection

CYFRA-21-1 has been recognised as a biomarker for cancer diagnostics, particularly for identifying non-small cell lung cancer. Scientists advanced the research in this domain by developing an electrochemical biosensor designed to detect CYFRA 21-1, employing differential pulse voltammetry (DPV) [114]. A biosensor [155] manipulated a nanocomposite foundation of reduced graphene oxide/indium tin oxide (rGrO/ITO), augmented with zirconia nanoparticles to increase sensitivity. This platform was further enhanced by functionalising with 3-aminopropyl triethoxy silane (APTES) and anti-CYFRA-21-1 antibodies. To address the issue of non-specific binding, BSA was integrated into the immunoelectrode composition. The biosensor exhibited an LOD at 0.122 ng$\;\cdot \;{\mathrm{mL}}^{-1}$ alongside a detection range of 2 to 22 $\mathrm{ng}\;\cdot \;{\mathrm{mL}}^{-1}$. The sensitivity of the device, expressed as the change in output signal relative to the change in analyte concentration, was determined to be 0.756 $\mu A\;\cdot \;\mathrm{mL}\;\cdot \;{\mathrm{ng}}^{-1}$. Moreover, the device presented stability, employing 94% of its operational ability after eight weeks of dry storage. The selectivity of the biosensor was evaluated against various biomarkers and potential interfering substances, including carcinoembryonic antigen (CEA) and cardiac troponin I (cTnI), among others. The results indicated interference, with a mean RSD of 0.32% and no notable effect was identified for detecting CYFRA-21-1d. Comparative analyses with enzyme-linked immunosorbent assay (ELISA), the conventional gold standard for biomarker detection, revealed a high correlation, with RSD values ranging from 0.2 to 3.35%. In a subsequent investigation, Kumar et al. introduced an alternative biosensor configuration, employing nanostructured metal oxides (NMOs) as cancer biosensors [156]. Although this device exhibited a slightly increased LOD and a more extensive linear range than its predecessor, it displayed a reduced stability of 90% after 27 days but considerably higher sensitivity at 18.24 $\mu A\;\cdot \;\mathrm{mL}\;\cdot \;{\mathrm{ng}}^{-1}$. The group utilised 2D electroactive rGrO to inhibit the Brownian motion of NMO, thereby reducing the cluster of NMO. Using nanostructured hafnium oxide (${\mathrm{nHfO}}_{2}$) as the model NMO and controlled hydrothermal synthesis, reduced agglomeration of ${\mathrm{nHfO}}_{2}$ was achieved. They investigated agglomeration inhibition through nanoparticle tracking analysis (NTA). To functionalise ${\mathrm{nHfO}}_{2}$ @rGrO, the researchers used APTES and deposited it onto an ITO-coated glass electrode using the electrophoretic deposition (EPD) technique. Furthermore, cancer biomarker antibodies (anti-CYFRA-21-1) were immobilised using EDC-NHS chemistry and blocked non-specific binding sites using BSA. The resulting immunoelectrode (BSA/anti-CYFRA-21-1/APTES/${\mathrm{nHfO}}_{2}$ @rGrO/ITO) demonstrated higher sensitivity (18.24 $\mu A\;\cdot \;\mathrm{mL}\;\cdot \;{\mathrm{ng}}^{-1}$), a broad linear detection range (0 to 30 ng$\;\cdot \;{\mathrm{mL}}^{-1}$), and a lower detection limit (0.16 ng$\;\cdot \;{\mathrm{mL}}^{-1}$) in the electrochemical response studies. The specificity of this device in detecting CYFRA-21-1 among similar interfering compounds was affirmed through additional testing against substances such as sodium bicarbonate (${\mathrm{NaHCO}}_{3}$), ascorbic acid, and lactic acid. Despite demonstrating a lower correlation with ELISA compared to the previous sensor model, with RSD ranging from 2.2% to 8%, the study validated the effectiveness of the new biosensor design. According to the authors, future research should concentrate on the development of more effective electroactive materials capable of detecting biomarkers across a wider spectrum.

Oral Cancer Detection

In 2017, Verma et al. developed an electrochemical immunosensor that utilises a composite of gold nanoparticles and reduced rGrO(AuNPs-rGO) as a transducer matrix. This method detected the salivary oral cancer biomarker IL8 without labelling or invasive procedures [157]. The combination of rGrO and AuNPs results in a rapid response and heightened sensitivity due to the enhanced electron transfer within the composite. The immunosensor detected IL8 in 9 min and had a range from 500 fg$\;\cdot \;{\mathrm{mL}}^{-1}$ to 4 ng$\;\cdot \;{\mathrm{mL}}^{-1}$, with a detection limit of approximately 72.73 ± 0.18 pg$\;\cdot \;{\mathrm{mL}}^{-1}$. This sensor demonstrated high recovery rates, selective detection among various substances, and remarkable reusability and stability, retaining over 91% functionality after four months of storage.

Verma and Singh improved this technology in 2019 by incorporating a zinc-oxide-reduced graphene-oxide (ZnO–rGrO) sensing platform with IL-8 antibodies, enhancing the sensor’s sensitivity and range [158]. Although the modified biosensor had a shorter evaluation period of 70 days, it showed reproducibility and stability in actual saliva samples, with recovery rates between 95% and 100%. After conducting tests, they discovered that the immunosensor could accurately detect IL8 at a low concentration range of 100 fg$\;\cdot \;{\mathrm{mL}}^{-1}$ to 5 ng$\;\cdot \;{\mathrm{mL}}^{-1}$ with a sensitivity of 12.46 ± 0.82 $\mu A\;\cdot \;\mathrm{mL}\;\cdot \;{\mathrm{ng}}^{-1}$ and a detection threshold of 51.53 ± 0.43 pg$\;\cdot \;{\mathrm{mL}}^{-1}$. The authors assert that the findings derived from the developed biosensor exhibit reproducibility and establish a prototype for the design of non-invasive biosensing platforms, thereby facilitating the detection of other cancer biomarkers through single- or multianalyte-based approaches in the future.

Prostate Cancer Detection

The glycoprotein enzyme prostate-specific antigen (PSA) is reported to be a biomarker for detecting prostate cancer, occurring in male serum at concentrations not exceeding 4 ng$\;\cdot \;{\mathrm{mL}}^{-1}$ [114]. Levels above this threshold require further diagnostic evaluations to exclude the presence of prostate cancer. Khan et al. introduced a chemo-resistive biosensor fabricated from graphene on Whatman filter paper (GrP-PS67-b-PAA27-Au), designed for the rapid and cost-effective detection of PSA in saliva [159]. Pure graphitic material improved electrical and thermal conductivity when applied to filter paper. It is reported that PS67-b-PAA27 acted as an amphiphilic connector between the GrP segments. By utilising the interaction between an anti-PSA antibody and a PSA antigen, this sensor creates a resistor within a circuit, resulting in a change in impedance that enables the detection and measurement of PSA levels in saliva samples. Real-time data was captured via a Bluetooth module, with a fully developed and refined device with a PSA detection range from 0.1 pg$\;\cdot \;{\mathrm{mL}}^{-1}$ to 100 ng$\;\cdot \;{\mathrm{mL}}^{-1}$. The sensor demonstrated a strong correlation coefficient of 0.963 and was capable of detecting prostate-specific antigen (PSA) at concentrations as low as 40 fg$\;\cdot \;{\mathrm{mL}}^{-1}$, allowing for identification well below clinically significant thresholds. It also displayed a linear response over a wide concentration range, maintaining a sensitivity of 0.875 $\Omega \;{\mathrm{fg}}^{-1}\;\mathrm{mL}$. Compared to earlier serum-PSA electrochemical biosensors, this saliva-PSA electrical biosensor demonstrates a quicker response time of 3–5 min. Its stability was verified for up to seven weeks in a dry environment, and its specificity was rigorously tested against beta 2-microglobulin, lactoferrin, and ascorbic acid. The integration of this sensor system with a compact handheld printed circuit board showcases its utility for point-of-care (POC) testing, further evidenced by a reproducibility rate of 88.89% across 72 identically produced sensors. Moreover, the sensor’s performance was cross-validated against a custom ELISA protocol, demonstrating a 94% concordance with this extensively recognised standard. The integration of electronic reading capabilities, validation through ELISA, and the achievement of a rapid response time ranging from one to three minutes establishes this graphene-based sensor as a vanguard prototype for future point-of-care (POC) biosensor technologies. With respect to future research directions, the authors propose that exploration into the design and optimisation of a compact miniaturised PCB integrated with a paper-based sensor should be pursued, as this configuration holds substantial promise for translation into a low-cost handheld platform. Such a device would not only possess significant commercialisation potential but also enable broader clinical adoption in point-of-care settings, thereby advancing accessibility and practical utility in healthcare.

According to Farzin et al., a voltammetric immunosensor was developed to detect PSA in saliva without the use of labels [160]. The sensor utilised a dual-purpose nanostructure of multiwalled carbon nanotube (MWCNT)/L-histidine-modified rrGrO(His-rGrO) to link the thionine redox indicator and anti-PSA antibody covalently. Incorporating MWCNTS enhanced the electrical conductivity and facilitated electron transfer to the GCE. However, the attachment of anti-PSA antibodies hindered thionine’s electron transfer, thereby reducing the redox signals. The immunosensor’s detection mechanism relied on the specific binding between PSA and the thionine-NH2-GO–COOH-Ab complex, resulting in a further reduction in the current signal of the electrochemical probe. The sensor had a linear calibration range of 10 fg$\;\cdot \;{\mathrm{mL}}^{-1}$ to 20 ng$\;\cdot \;{\mathrm{mL}}^{-1}$, with a correlation coefficient 0.996. Under optimal conditions, the immunosensor could detect PSA with a detection limit of 2.8 $\mathrm{fg}\;\cdot \;{\mathrm{mL}}^{-1}$ and displayed enhanced recovery rates in [159]. However, it lacks an integrated readout system, requiring a connection to an electrochemical analyser for data interpretation. Its long-term stability was affirmed at 97% and 94.7% after two and four weeks, respectively, with its selectivity rigorously evaluated against various interfering substances. Reproducibility was assessed within and between assays, yielding RSD values of 2.9% and 5.7%, respectively. When validated against the ELISA gold standard, the biosensor exhibited a 97.6% accuracy rate, affirming its efficacy for PSA screening in saliva compared to conventional methodologies. The authors emphasise the platform’s adaptability to point-of-care approaches, suggesting the development of portable, user-friendly devices for rapid, non-invasive prostate-specific antigen detection in real-world applications.

Table 4 and Table 5 present the features of graphene-based, non-invasive saliva-sensing devices for detecting viruses and biomolecules. For saliva-based diagnostics, which are primarily aimed at rapid, point-of-care testing of low-concentration biomarkers like hormones, viruses, and proteins, the criteria used to evaluate the performance of the sensors in Table 4 and Table 5 are:

1. Sensitivity: A signal change of ≥10% across the pM–nM range for clear signal-to-noise in dilute samples.
2. Detection Range: The ability to span the clinically relevant picomolar (pM) to nanomolar (nM) concentration range.
3. Limit of Detection (LOD): A stringent threshold of <100 pg/mL, essential for detecting trace analytes for early-stage disease diagnosis (<100 pg/mL for proteins/viruses or <1 μM for metabolites).
4. Response Time: A rapid result time of <5 min (300 s) to enable point-of-care use.
5. Durability: A minimum storage stability of >30 days, which is a critical parameter for the commercial viability of disposable test kits. This addresses shelf stability and requisite operational lifespan for disposable or semi-disposable systems.
6. “U” values are conservatively deemed non-compliant to mitigate overestimation risks

This benchmarking approach allows a rigorous head-to-head comparison across diverse analytes, including viral antigens (e.g., HPV-16, SARS-CoV-2), cancer protein biomarkers (e.g., PSA, CYFRA-21-1, IL-8), metabolic analytes (e.g., glucose, uric acid), and small molecule hormones (e.g., cortisol), highlighting both promising candidates and translational bottlenecks.

Table 4 and Table 5 demonstrate that the majority (81%) of the reviewed devices meet three or fewer of the five benchmarks, underscoring the translational gap between laboratory demonstrations and clinically deployable devices, indicating that most reported systems remain far from practical point-of-care viability. However, the potential of this field is underscored by a select group of high performers. The GrP/PS67-b-${\mathrm{PAA}}_{27}$-Au sensor for PSA meets all five criteria, while only six (16%) of all reviewed systems achieved a strong score of 4/5, demonstrating an excellent balance of sensitivity, speed, and stability. These high-performing systems illustrate the feasibility of saliva-based platforms capable of satisfying stringent clinical benchmarks, sharing common design features such as hybrid nanocomposites, stable immobilisation chemistries, and miniaturised device formats that provide a roadmap for next-generation diagnostics.

Detection ranges are generally well aligned with clinical requirements, but a clear trade-off emerges between sensitivity and speed. Affinity-based sensors for proteins and viruses consistently achieve excellent LODs in the pM–fM range, yet sacrifice response time, with nearly 87% of devices failing to deliver results within the <5 min benchmark. Sensitivity reporting is also inconsistent, with many studies omitting or failing to meet the ≥10% signal change threshold, raising concerns about reliability in complex salivary matrices. Durability is variably addressed, with some devices demonstrating weeks of stability, but few are validated under accelerated aging or real-world humidity and temperature stress, a critical gap for commercial viability.

These findings highlight that while sensitivity and detection range are often achieved, the next step for the field is to translate this analytical performance into devices that are not only accurate but also rapid and stable. Sensors achieving 4–5/5 compliance represent strong candidates for near-term translation, while those scoring 0–3/5 underline persistent challenges in assay robustness and reproducibility. Importantly, the heterogeneity in reporting practices (e.g., frequent “U” entries) signals the urgent need for standardised performance evaluation protocols. Addressing these issues through harmonised testing, accelerated stability studies, and multiplexed assay formats will be critical for advancing saliva sensors from laboratory prototypes into robust, deployable point-of-care platforms.

#### 2.1.3. Tear Biomarker Detection

Tears are produced by the lacrimal gland. It contains proteins, enzymes, lipids, electrolytes, metabolites, and mostly water (over 98%) [161,162,163]. These components serve as biomarkers that offer valuable insights into systemic diseases and ocular conditions [164,165,166]. Many tear biomarkers are closely linked to blood biomarkers, with glucose levels in tears highly correlated with blood glucose levels [167,168,169]. Because of low secretion rates, non-uniform production, and complex collection procedures, it can be challenging to analyse tear samples [142,170,171]. However, wearable tear-sensing devices, such as contact lens-based platforms, can overcome these issues [172,173]. These devices integrate biosensing elements, transducers, data processing, and power sources and are particularly suitable for tear collection as they do not cause eye irritation or damage. Graphene-based contact lenses are especially noteworthy, as they offer better biocompatibility, minimal eye irritation, and no discomfort or damage to the wearer.

##### Glucose Detection

A study conducted by Kim et al. [46] proposed a wearable contact lens biosensor made of graphene–silver nanowire (Gr-AgNW) hybrid networks. The biosensor wirelessly detected glucose concentrations from 1 μM to 10 mM, making it a tool for monitoring disease-related markers and assessing overall health. In addition, the biosensor was reported to be highly transparent and stretchable, offering clear vision alongside exceptional comfort for the wearer. An RLC circuit was also integrated into the contact lens, enabling the simultaneous and independent monitoring of tear fluid composition and intraocular pressure. The paper emphasises that further advancements in sensor technology are requisite for accurate glucose diagnostics. They additionally note that their innovative pyrene–graphene chemistry could be adapted to facilitate the selective detection of a broader array of biomarkers, thereby indicating potential avenues for future expansion beyond glucose monitoring.

Park et al. [174] conducted a study introducing a soft contact lens designed to detect glucose concentration in tears in real time using wireless technology. The glucose sensor in the lens was made of graphene and was immobilised with catalase (CAT) and glucose oxidase (GOx). The lens featured a stretchable, transparent antenna and rectifier, enabling the LED display to turn on and provide real-time sensing results. It presented a detection limit of 12.57 μM and detected glucose concentrations within 0.1∼0.9 mM. The lens had a response time of approximately 1.3 s, with a sensitivity of about 22.72% ${\mathrm{mM}}^{-1}$. The researchers explicitly advocate that future developments should concentrate on enhancing the design of the smart contact lens system to provide users with more intuitive readouts. Specifically, they propose integrating biomarker monitoring through a smartphone interface. This direction underscores the importance of usability and seamless integration with portable electronic devices as their projected next step.

##### Cytokines Detection

Wang et al. developed a wearable aptasensor based on GFET technology for detecting the inflammatory cytokines TNF-α and IFN-γ in an artificial tear matrix [175]. The device utilised graphene functionalised with specific aptamers for biorecognition. To create this biosensor, they used an ultrathin mylar film substrate and a lithography technique to pattern the electrodes, with the conducting channel being formed by transferring the graphene sheet onto the electrodes using a polymethyl methacrylate (PMMA) carrier layer and dissolving the PMMA layer with acetone. The sensor was biochemically functionalised using 10 mM 1-pyrenebutanoic acid succinimidyl ester (PASE) solution. The ultrathin substrates were implemented to enhance the mechanical stability of biosensors, enabling their integration onto curved surfaces such as human skin or eyeballs. The biosensor demonstrated sensitivity in detecting cytokines TNF-α and IFN-γ, with detection limits down to 2.75 pM and 2.89 pM, respectively. This manuscript lacks a dedicated “Future Work” section; however, the authors provide insights into their wearable GFET biosensor’s potential and suggest avenues for enhancement through their performance discussion. The authors view their work as a foundational step towards broader applications, indicating the necessity of further development for widespread practical adoption. They highlight the biosensor’s sensitivity, specificity, and performance under significant deformations, while also identifying areas for improvement. These include enhancing integration into wearable systems, expanding the range of detectable biomarkers, and optimising functionality in complex biofluids. Additionally, the biosensor’s ability to conform to non-planar surfaces, such as human skin and the eyeball, and withstand substantial tensile strains suggests directions for advancing mechanical durability and scalability for practical applications.

##### Ocular Detection

In 2021, Jang et al. unveiled a landmark study in biotechnology and ophthalmology, documenting the first human trial of a healthcare solution combining a soft, intelligent contact lens with a skin-compatible, wearable therapeutic device [176]. The core diagnostic feature of this system was a luminous contact lens engineered to facilitate the in vivo, real-time quantification of matrix metalloproteinase-9 (MMP-9) concentrations in tear fluid, a critical biomarker for chronic ocular surface inflammation (OSI). This capability was achieved by integrating a GFET within the lens, a pioneering approach in ocular diagnostics. The graphene surface of GFET was functionalised with immunoglobulin G (IgG). AgNWs were implemented to provide conductive pathways. It was characterised by an LOD of 0.74 ng$\;\cdot \;{\mathrm{mL}}^{-1}$ and a detection spectrum ranging from 1 to 500 ng$\;\cdot \;{\mathrm{mL}}^{-1}$. Furthermore, it came with an eyelid-attachable heat patch for hyperthermia treatment, allowing for both diagnosis and continuous treatment of ocular scatter index OSI. The device’s reliability and wearability for sustainable personal health management have been confirmed through in vivo studies on human subjects and live animals. The text asserts that future research should encompass clinical analyses that correlate chronic ocular surface inflammation (OSI) levels in tears with other biomarkers under varying physiological conditions. The authors also highlight the design’s potential for the incorporation of machine learning techniques to augment disease diagnostics.

##### Myopia Detection

Zhang et al. developed an electrochemical biosensor that monitored myopia by detecting dopamine levels in tears [177]. This biosensor was produced using electroactive nanoelectrodes from poly (3,4-ethylene dioxythiophene) functionalised sulphur-doped graphene (PEDOT-Gr), created through a vapour phase polymerisation (VPP)-enhanced ball milling method. The enzyme tyrosinase receptor is then covalently immobilised onto the thin PEDOT-graphene nanosheets. The result is a biosensor that is electrodeposited into the shape of a wearable corneal sensor that fits the eye’s structure. The biosensor demonstrated a sensitivity of $12.9\times 10^{-3}$$\mu A\;\cdot \;M^{-1}\;{\mathrm{cm}}^{-2}$ and a detection limit of $101\times 10^{-9}$ M. Myopia patients induced by defocus were tested using the corneal biosensor, and the results showed a relationship between myopia diopters and DA content in tears. These findings suggest that the corneal biosensor has great potential for early and real-time detection and prevention of myopia. The authors advocate for the refinement of their PEDOT-functionalised sulphur-doped graphene corneal biosensor by developing multiplexed sensing capabilities for additional biomarkers, such as dopamine, which would enhance diagnostic precision for myopia progression. They further recommend optimising the PEDOT:S-graphene composite to improve both sensitivity and mechanical flexibility, in addition to integrating the sensor with smart contact lens platforms or mobile health systems to enable real-time monitoring. Moreover, they discuss conducting large-scale clinical trials and developing scalable fabrication processes to validate performance across diverse populations, ensuring cost-effective production for broader clinical adoption.

##### L-Cysteine Detection

**Figure 11:**
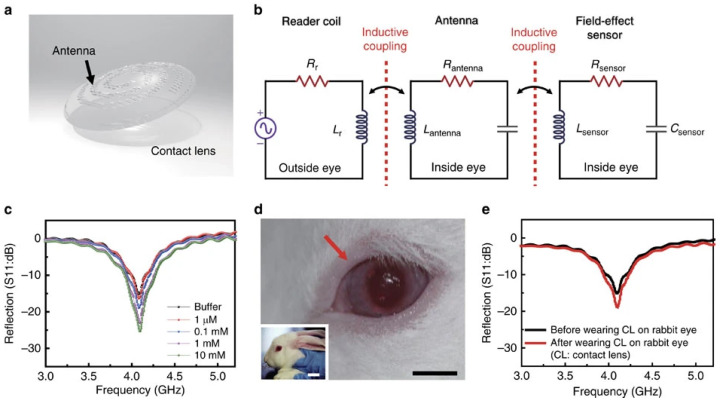
(**a**) Design schematic of a transparent, contact lens-integrated glucose sensor. (**b**) Circuit architecture enabling wireless readout. (**c**) Calibration curve for wireless glucose detection (1 μM–10 mM). (**d**) In vivo deployment on a live rabbit; scale bars: 1 cm (black), 5 cm (white). (**e**) Temporal glucose response profiles pre- and post-lens application in the same animal model. (Reproduced from [46] with permission from Nature).

**Figure 12:**
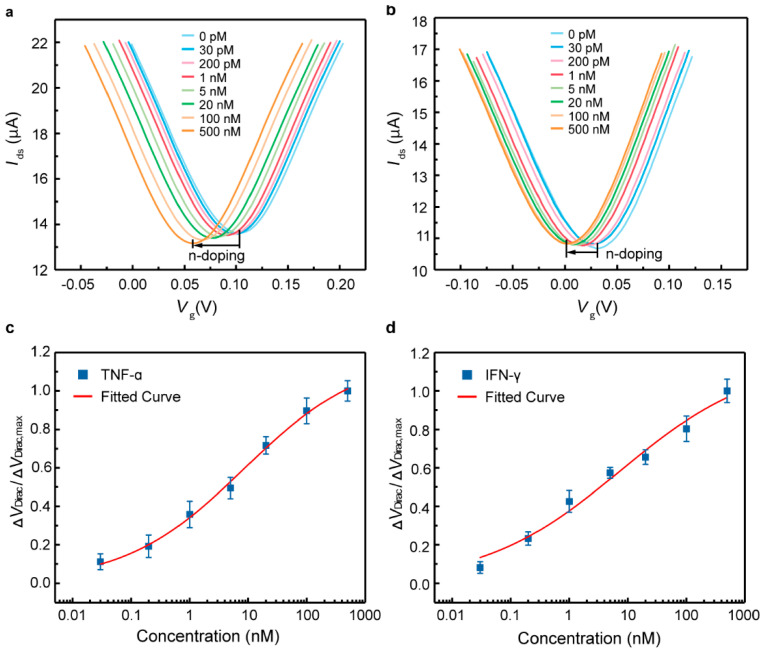
Cytokine detection in simulated tear fluid. Transfer characteristics recorded upon exposure to (**a**) TNF-α and (**b**) IFN-γ across a concentration gradient. (**c**,**d**) Normalised Dirac point shifts plotted against cytokine concentration, fitted using the Hill–Langmuir model. (Reproduced from [175] with permission from MDPI).

Huang et al. presented a wearable nanosensor to detect biomarkers in body fluids [107]. This nanosensor was made from an ultra-flexible and transparent GFET, which incorporates a receptor designed to attach specifically to a biomarker. When connected, the receptor triggers a visible shift in the graphene’s carrier concentration. This nanosensor is remarkable because it can undergo 100 deformation cycles without visible damage, including bending at 175 μm radii, folding at 150 °C, and shrinking by 50%. Despite these deformations, its electrical properties remain highly stable. The sensor’s ability to detect L-cysteine, a biomarker linked to various health conditions, with an LOD of $0.043\times 10^{-6}\;m$ in artificial tears, showcases its capabilities. Various concentrations of L-cysteine in artificial tears, ranging from 0 to $4800\times 10^{-6}\;m$, have been effectively detected by this nanosensor. The sensor uses a 1 μm-thick layer of transparent polyethene glycol terephthalate and a tungsten trioxide/Gold/Tungsten trioxide (${\mathrm{WO}}_{3}$/Au/${\mathrm{WO}}_{3}$) electrode with 81% transparency. It would be placed on the eyeball for tear analysis without obstructing vision. These findings suggest that the GFET wearable nanosensor has significant potential for medical diagnostic applications. Figure 11 and Figure 12 and Table 6 present different graphene-based non-invasive tear sensors and their features that detect different biomolecules such as glucose, cytokines, dopamine, etc. In discussing future directions, the authors indicate that such devices can be improved by creating highly integrated and minimally invasive pl atforms that allow for the multiplexed and real-time detection of multiple biomarkers concurrently. They also emphasise the importance of ensuring biocompatibility for safe on-skin and ocular applications, recommending the integration of GFET sensors with wireless data transmission modules to facilitate truly continuous health monitoring in practical, real-world situations.

Tear-based biosensors, often envisioned for integration into smart contact lenses for continuous monitoring, face unique challenges related to very low sample volume and the need for high biocompatibility. The performance of the devices in Table 6 was assessed against benchmarks tailored for this context:

1. Sensitivity: ≥10% signal change.
2. Detection Range: Must cover relevant physiological ranges (e.g., 0.1–0.9 mM for glucose, pM cytokines).
3. Limit of Detection (LOD): A threshold of <15 μM for glucose (critical for hypoglycemia detection) and <1 nM for other biomarkers.
4. Response Time: A very rapid response of <30 s is required for real-time physiological feedback.
5. Durability: A minimum of >12 h of continuous operational stability to ensure reliability for a full day of wear.

We treated “U” as failures for methodological transparency.

The analysis of tear-based graphene sensors highlights their potential for non-invasive monitoring, with Gr/CAT/GOx for glucose emerging as a leader, meeting four of five benchmarks (sensitivity >$10\%\;{\mathrm{mM}}^{-1}$, 0.1–0.9mM range, 1.3s response, 48h durability) despite a 12.57μM LOD exceeding the <5μM threshold. GFET-based cytokine sensors (e.g., TNF-α at 2.75pM) achieve sub-nM LODs but lag with >7min responses and unverified ocular stability, while the L-cysteine GFET offers mechanical robustness (100 cycles) yet lacks sensitivity validation in real tears. Fast response times (<30s in 2/6 studies) and strong durability (4/6>24h) underscore kinetic and structural advances, though the field’s small sample (6 studies) reveals engineering challenges over fundamental sensing limits. A sensitivity–speed–durability trilemma persists: enzymatic electrodes excel in kinetics, FETs in LOD, but few integrate all. Contact lens multiplexing remains underexploited, necessitating biocompatible power solutions and long-term stability studies for translational success.

### 2.2. Breath Sensing Devices

Exhaled breath is another source that can be used to detect various biomarkers and moisture content [59]. Human breath comprises many compounds, including $N_{2}$, $O_{2}$, ${\mathrm{CO}}_{2}$, and water vapour. In addition, several volatile organic compounds (VOCs) [178], such as acetone, ethanol, and CO, can offer insight into various medical conditions, including diabetes [179,180], and cancer [181]. Specialised graphene sensors can analyse, breathe and pinpoint specific VOCs to diagnose these conditions [182].

#### Volatile Organic Compounds (VOCs) Detection

Xu et al. explored the use of graphene in biosensors for detecting VOC biomarkers in breath and identifying various physiological signals [182]. Their study presented multifunctional sensing devices utilising porphyrin-modified rGrO arrays as the sensor, allowing for monitoring volatile compounds in breath and a strain sensing matrix with porous rGrO films to detect physiological signals. The sensor successfully differentiated between different human physiological signals and various VOCs in breath, making it useful for wearable health monitoring of an individual. The sensor was tested on simulated breath samples with diabetes, nephrotic breath samples, and breath samples of a healthy individual. Based on the output pattern, the patient’s condition was determined. The paper does not include an explicit “future work” section. From the discussion, it can be inferred that future efforts should focus on improving the long-term mechanical and chemical stability of the graphene films, validating performance under continuous wear, and integrating compact wireless readout modules.

Liu et al. developed an electronic nose that detected VOCs and cancer biomarkers such as ethanol, 2-ethylhexanol, nonanal, and ethylbenzene with high sensitivity (25 ppm) [183]. The sensor used rGrO functionalised with eight amine molecules, providing additional adsorption capacity for VOCs. It offered a linear response to detecting cancer biomarkers, making it ideal for wearables. The device had a speedy response time for detecting acetones in breath, which was an easy method for diabetes detection. The sensor also exhibits selectivity towards acetone, with chitosan having several -${\mathrm{NH}}_{2}$ groups that remain exposed to interact specifically with the carbonyl groups in acetone. The device’s sensing capability is tested under different acetone concentrations, and it shows a quick response time of less than a second with a small detection limit of 20 ppb. These authors highlight humidity tolerance, ultralow VOC detection, and improved material functionalisation as the key priorities for future work, providing a clear roadmap towards clinical applicability.

In 2020, Su et al. introduced an innovative non-invasive wearable biosensor designed for prediabetes detection [184]. The sensor integrated chitosan (CS) and rGrO, comprising a power-generating unit and a sensing component. It leveraged triboelectric effects and chemisorption to function in a wearable format. Energy was generated through periodic contact between polytetrafluoroethylene (PTFE) and nylon films, driven by airflow-induced vibrations. At 97.3% relative humidity and ambient temperature, the CS-rGrO composite sensor exhibited a 27.89% response to 10 ppm acetone in exhaled breath, outperforming CS-only sensors. Finite element analysis and phase-field simulations were used to delineate the sensing mechanism. However, the sensor’s performance was evaluated using simulated samples rather than human breath. While the article emphasises proof-of-concept performance, it does not provide explicit statements of future work. The work states that the sensor shows *“a promising capability of practical respiratory monitoring”*. The authors conclude by proposing that their work *“paves the way for a new method of acetone sensing in respiration and non-invasive prediabetes diagnosis both experimentally and theoretically”.*

Sanchez-Vicente et al. developed an advanced sensor composed of pristine graphene-doped tin oxide ($\mathrm{PGr}-{\mathrm{SnO}}_{2}$) that can detect a range of gases, including ethanol, acetone, nitrogen oxide (NO), and carbon monoxide (CO), present in human breath [185]. These gases indicate various health conditions, such as chronic obstructive pulmonary disease, asthma, cystic fibrosis, and diabetes. The sensor exhibited the highest sensitivity to acetone and ethanol throughout all temperature ranges, with a detection efficiency of over 35%. However, it was less effective in detecting CO and NO at lower temperatures, only becoming more efficient at temperatures above 100 °C. The sensor detected the mentioned biomarker gases at concentrations typical for healthy individuals and those suffering from various illnesses, with ideal detection performance at 300 °C. The sensor also operated effectively at room temperature, particularly for acetone and ethanol detection. Environmental testing revealed that increasing the relative humidity from 0% to 50% decreased the response to ethanol and acetone but, conversely, enhanced the sensitivity towards CO and NO, highlighting the critical role of ambient moisture in sensor performance. Graphene in the sensor’s design notably improved its performance in low-temperature environments and overall gas detection capabilities by forming an n-p heterojunction. The detection abilities of the sensor were tested using ethanol concentrations ranging from 0.5 to 2 ppm, acetone from 0.5 to 4 ppm, CO from 1 to 5 ppm, and NO from 10 to 100 ppb. However, these tests were only performed using simulated breath. Therefore, further testing is required in real-world scenarios. The paper sets out a roadmap focusing on humidity testing, energy-efficient non-heated substrates, and material diversification to enhance selectivity and enable future developments. It envisions portable, low-cost equipment for health centres or home use, potentially integrated into wearables, gadgets, smartphones, or smartwatches.

Tuan et al. developed a method to improve the sensitivity and specificity of chemoresistive sensors, which are used to detect VOC biomarkers [186]. They achieved this by combining PGr with MOFs, resulting in a nanocomposite with a synergistic effect. It was reported that the combination provided high conductivity through graphene, while MOFs offered a large surface area and adsorption capability and improved detection abilities. The sensor’s performance can be customised for specific VOC biomarker identification by carefully selecting different MOFs. The study examined the performance of graphene hybrid nanocomposites with MOFs such as copper benzene-1,3,5-tricarboxylate (PGr-Cu BTC), zirconium 1,4-dicarboxybenzene (PGr-UiO 66), and 2-methylimidazole zinc salt (PGr-ZIF 8) in identifying various VOCs, including methanol, ethanol, chloroform, acetone, acetonitrile, and THF. The PGr-Cu BTC nanocomposite exhibited the most considerable response to methanol, ethanol, and chloroform, particularly to chloroform, followed by methanol and ethanol, while showing lower responses to acetone, acetonitrile, and THF. The PGr-Cu BTC sensor demonstrated superior sensitivity and selectivity, especially to chloroform and methanol VOCs, with detection levels ranging from 2.82 ppm to 22.6 ppm, making it the most effective graphene-MOF sensor tested. However, PGr-UiO 66 responded reasonably well to methanol and chloroform, but was lower than PGr-Cu BTC. The manuscript’s concepts offer a valuable approach for developing low-cost, high-performance VOC biomarker sensors for human health monitoring, with further potential for non-invasive, personalised telehealth diagnostics. By linking these sensors wirelessly via the Internet of Things (IoT), they could be implemented as cost-effective and efficient tools for point-of-care (PoC) and telehealth monitoring.

**Figure 13:**
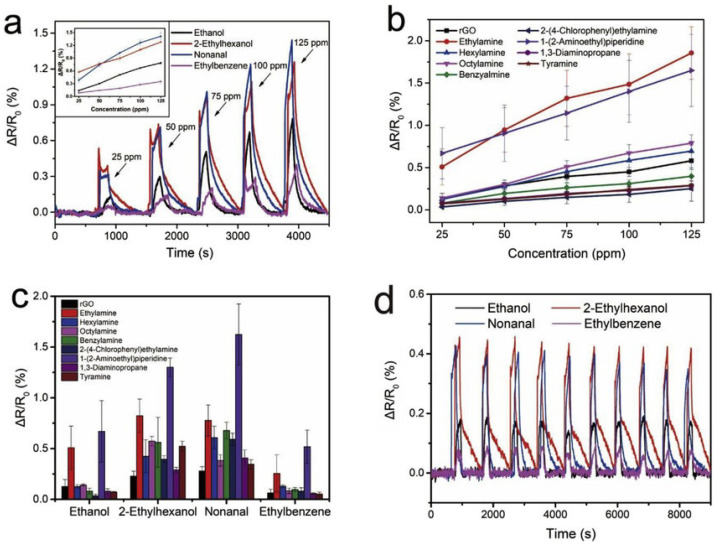
(**a**) Dynamic response of octylamine-rGO sensing elements to different VOC levels; inset highlighting linear concentration dependence. (**b**) Variation in responses to ethanol across nine sensing elements. (**c**) Comparative behaviour across four VOCs (25 ppm). (**d**) Repeatability across 10 cycles at 25 ppm VOC (ethanol). (Reproduced from [183] with permission from ScienceDirect).

**Figure 14:**
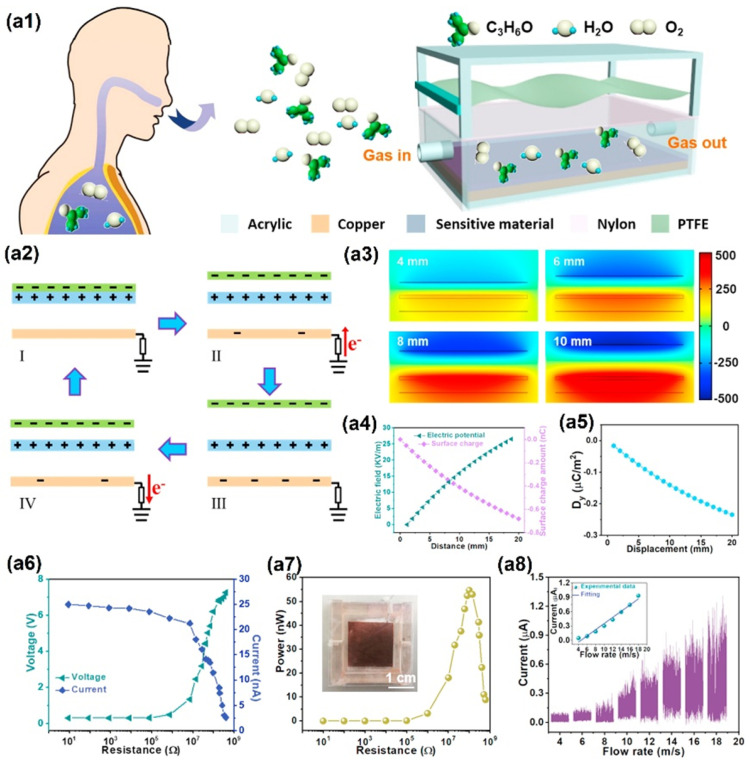
Wireless power-assisted acetone sensor (WSAS). (**a1**) Conceptual diagram of breath acetone recognition. (**a2**) Illustration of energy harvesting mechanism. (**a3**–**a5**) Finite element predictions of potential distribution, electric field, charge transfer, and displacement between PTFE/nylon films. (**a6**,**a7**) Device output characterisation with resistance loadings, including generated voltage, current, and power (inset: image of fabricated sensor, scale bar: 1 cm). (**a8**) Current output as a function of breath flow rate with average current shown in the inset. (Reproduced from [184] with permission from ScienceDirect).

**Figure 15:**
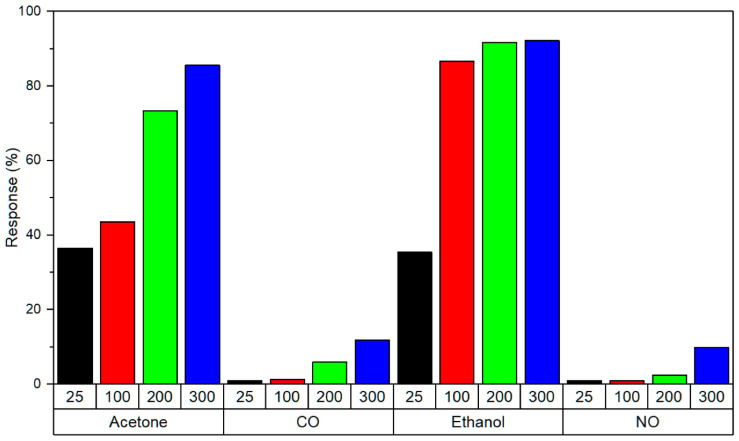
Temperature-dependent sensor responses to 2 ppm ethanol, 4 ppm acetone, 5 ppm CO, and 100 ppb NO, evaluated at 25, 100, 200, and 300 °C. (Reproduced from [185] with permission from MDPI).

**Figure 16:**
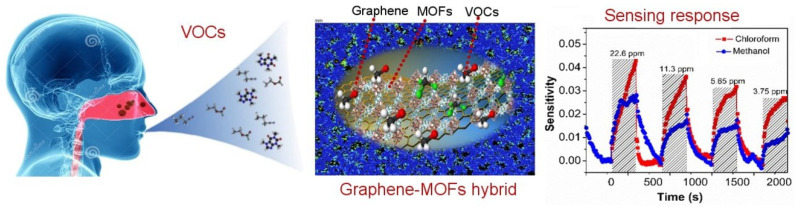
Conceptual schematic of detecting VOC biomarkers related to human health using graphene–MOF hybrid composites. (Reproduced from [186] with permission from ScienceDirect).

**Figure 17:**
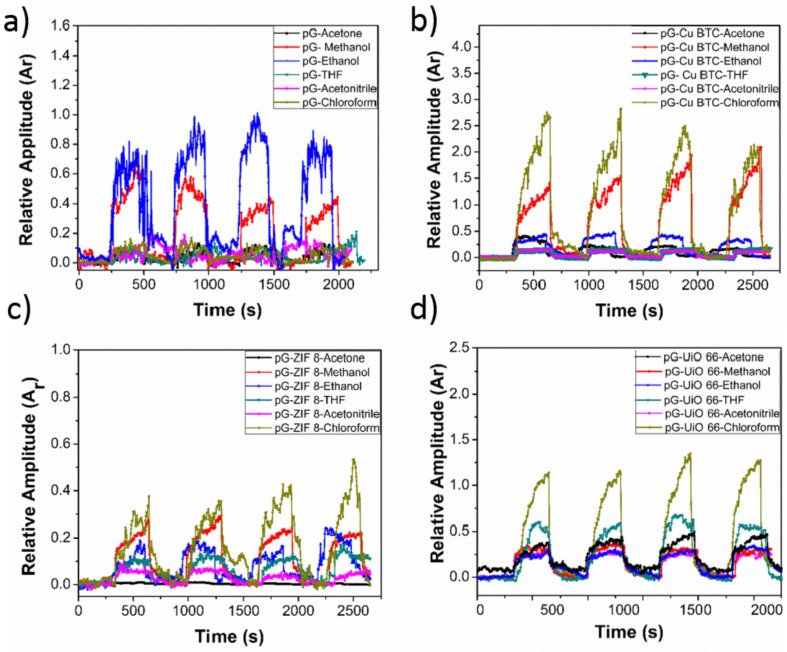
Comparative sensing performance between pristine graphene and graphene–MOF hybrid sensors for saturated vapour concentrations of (**a**) pristine graphene, (**b**) pG-Cu BTC, (**c**) pG-ZIF-8, and (**d**) pG-UiO-66. (Reproduced from [186] with permission from ScienceDirect).

Figure 13, Figure 14, Figure 15, Figure 16 and Figure 17, and Table 7 show different graphene-based non-invasive sensors and their features, respectively, that detect VOC in breath.

Breath sensing targets volatile organic compounds (VOCs) at trace levels (ppb–ppm), requiring resilience to high humidity, and presents a unique opportunity for truly non-contact diagnostics. The benchmarks for the sensors in Table 7 are defined by the specific challenges of this medium with “U” conservatively interpreted as non-meeting to uphold analytical rigor:

1. Sensitivity: ≥10% signal change.
2. Detection Range: Spanning the relevant parts-per-billion (ppb) to low parts-per-million (ppm) range.
3. Limit of Detection (LOD): A highly sensitive threshold of <100 ppb is necessary to distinguish pathological VOC levels from healthy baselines.
4. Response Time: A rapid response of <60 s to capture transient breath components.
5. Durability: Demonstrated stability in the face of the primary interferent, high humidity, defined here as stable operation at ≥80% Relative Humidity (RH).

The benchmarking of breath sensors, the most nascent of the four biofluid fields, reveals no device satisfies all criteria, highlighting limitations in real-world applicability. Several sensors, notably PGr-${\mathrm{SnO}}_{2}$, demonstrate rapid response times (<60 s) and cover clinically relevant detection ranges (1 ppb–10 ppm), but most (3/5) fall short of the LOD benchmark (<100 ppb), with only PGr-${\mathrm{SnO}}_{2}$ achieving compliance (0.01–0.5 ppm). A systemic lack of durability data, particularly under ≥80% RH, remains a critical gap, with all entries unreported. PGr-${\mathrm{SnO}}_{2}$ leads with 3/5 benchmarks, excelling in sensitivity (>35%) and speed. The field faces a materials challenge: achieving room-temperature, humidity-resistant selectivity at ppb levels. Chemiresistive graphene–MOF hybrids (e.g., PGr-Cu BTC) show promise but require validation in human breath beyond synthetic mixtures. Future research must enhance sensitivity to <100 ppb and develop robust, humid-stable systems for reliable exhaled breath monitoring.

### 2.3. Breath Comparative Insights

Breath sensors (Table 7) predominantly exploit chemiresistive (CR) and electrochemical (EC) detection of volatile organic compounds (VOCs) and gases such as acetone, ethanol, ${\mathrm{NH}}_{3}$, NO, and CO. Porous graphene–metal oxide hybrids (e.g., PGr–${\mathrm{SnO}}_{2}$, PGr–CuBTC) deliver high sensitivity in the ppm–ppb range, benefiting from synergistic catalytic activity. However, they often require elevated operating temperatures, increasing power consumption and limiting wearability.

Chemiresistive chitosan–rGO sensors achieve good selectivity towards acetone at room temperature but show cross-sensitivity to humidity—a major confounder in exhaled breath analysis. In contrast, functionalised rGO electrodes provide wider analyte coverage but at the cost of lower selectivity.

Collectively, graphene-based breath sensors demonstrate promise for non-invasive monitoring of diabetes (acetone), liver function (ethanol), and air quality exposure (NO, CO). To move towards clinical translation, future work must focus on room-temperature operation, humidity compensation, and integration with machine-learning-driven e-nose arrays to achieve reliable multiplexed detection in real-world breath samples.

## 3. Unified Benchmarking of Biofluid Platforms

**Figure 18:**
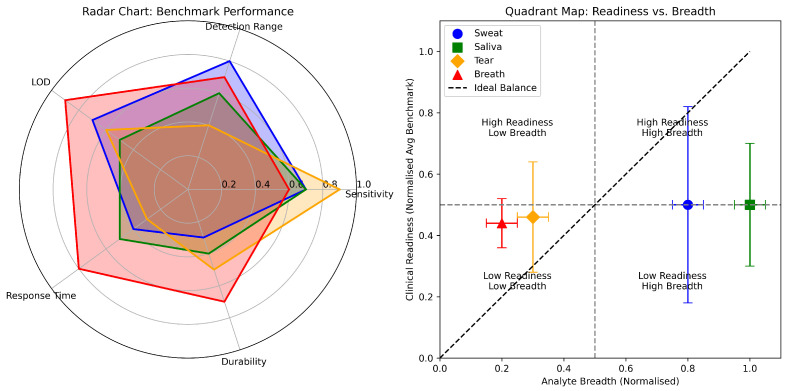
A multi-dimensional comparison of the translational readiness of non-invasive biofluid sensors. (**Left**) Radar chart depicts individual benchmark performance (sensitivity, detection range, limit of detection [LOD], response time, durability) scored from 0 to 1. (**Right**) The quadrant map visualises the broader translational landscape by plotting clinical readiness against analyte breadth for all sensors. The vertical axis represents technological maturity (average benchmark score), and the horizontal axis represents analyte versatility. Together, the panels illustrate that while sweat has the highest-performing individual devices, saliva exhibits a more broadly mature profile. In contrast, tear and breath are more nascent, specialised fields, with breath being the most homogenous but least mature, underscoring the different developmental challenges and strategic opportunities across the non-invasive sensing landscape. Data estimated from Table 3, Table 4, Table 5, Table 6 and Table 7.

To consolidate the biofluid-specific evaluations, sweat, saliva, tear, and breath platforms were integrated into a unified benchmarking framework (Table 8 and Figure 18), enabling direct comparison of translational maturity and performance consistency. The analysis reveals distinct developmental trajectories shaped by physiological constraints and engineering priorities, while also exposing recurring limitations. This synthesis provides a robust foundation for advancing graphene-enabled diagnostics towards clinical translation.

Sweat has emerged as the most advanced domain, reflecting both high research intensity and the presence of devices capable of meeting all five benchmarks. Platforms optimised for dynamic monitoring during physical activity demonstrate strong mechanical robustness and rapid response, spanning the widest performance spectrum from early prototypes to fully compliant wearable systems. Four devices achieved perfect 5/5 compliance. Yet variability remains high (SD ≈ 1.6), with half of the devices meeting only one benchmark. This fractured, bimodal landscape underscores both exceptional promise and persistent reproducibility gaps, highlighting the urgent need for standardised validation to reduce disparities in durability reporting, response times, and low-concentration metabolite detection.

Saliva biosensors exhibit moderate variability (SD ≈ 1.0), with most devices clustered at 2–3/5 compliance (≈65%). This reflects balanced but heterogeneous progress: many platforms achieve excellent analytical sensitivity and clinically relevant LODs, yet these gains are consistently offset by slow assay kinetics, variable response times, and inconsistent stability validation. Six devices achieved 4/5 compliance, demonstrating the field’s diagnostic breadth, particularly for proteins and viruses. However, the translational impact of saliva sensing remains constrained by reproducibility gaps and heterogeneous reporting practices, limiting its readiness for point-of-care deployment.

Tear biosensors display low variability (SD ≈ 0.9), though this partly reflects the small evidence base. Most devices cluster at 2/5 compliance (≈50%), underscoring persistent challenges in balancing sensitivity, speed, and durability. Several platforms achieve picomolar cytokine detection and sub-thirty-second response times, combining enzymatic electrodes with ultra-low LOD FET architectures. Yet translation is hindered by limited validation in real ocular environments, insufficient durability data, and the intrinsic microvolume of tears, which constrains reproducibility. Concentrated performance around moderate scores highlights the need for microfluidic and biocompatibility innovations to enable reliable continuous monitoring.

Breath remains the least mature domain, with the lowest variability (SD ≈ 0.4) and nearly all devices clustered at 2/5, plus a single platform at 3/5. This narrow dispersion signifies a homogeneous but uniformly modest performance profile. Fundamental materials challenges, particularly humidity resilience and the inability to achieve sub-100 ppb detection limits for volatile organic compounds, continue to constrain progress. The absence of high-performing outliers underscores the immaturity of the field and the urgent need for humidity-resistant sensing architectures and advanced materials engineering to enable meaningful clinical translation.

These divergent profiles underscore that no single biofluid offers a universal solution; rather, each occupies a distinct niche defined by trade-offs between sensitivity, speed, stability, and practicality. Table 8 consolidates these findings into a cross-biofluid benchmarking summary, facilitating transparent device comparisons and highlighting that progress is unevenly distributed. Crucially, technological maturity is not simply a function of research duration but depends on how effectively platforms address biofluid-specific operational challenges. Converting analytical promise into translational readiness will therefore require targeted engineering strategies for each biofluid, underpinned by harmonised benchmarking protocols to ensure reproducibility and comparability. Overall, sweat and saliva offer broader analyte versatility for wearable and point-of-care applications, whereas tear and breath hold promise for specialised continuous or non-contact monitoring, underscoring field-wide needs for improved durability and standardised reporting.

Clinical translation will depend on biofluid-specific engineering strategies coupled with harmonised benchmarking. For sweat, priorities include achieving trace-level metabolite LODs and standardised durability validation; for saliva, accelerating assay kinetics through improved bioreceptor design and microfluidics; for tears, advancing ultra-low volume handling and long-term ocular biocompatibility; and for breath, developing humidity-resistant nanomaterials with ppb-level selectivity. Equally critical is the adoption of community-wide reporting standards that eliminate incomplete datasets and mandate physiologically relevant testing. By aligning innovation with rigorous, transparent validation, non-invasive biosensors can progress from promising prototypes to robust, deployable platforms for precision diagnostics and continuous health monitoring. Ultimately, the future of non-invasive biofluid sensing lies in transforming isolated high-performance demonstrations into reproducible, clinically validated platforms that redefine continuous health monitoring and precision diagnostics.

## 4. Challenges and Limitations in Graphene-Based Biosensor Development

Graphene’s exceptional electrical, mechanical, and thermal properties make it an attractive candidate for developing non-invasive biochemical sensors aimed at human health monitoring. Such sensors are designed to detect biomarkers, e.g., glucose, lactate, cortisol, or electrolytes in sweat, saliva, tears and breath without requiring invasive procedures, e.g., blood extraction. However, despite the promising potential of graphene-based sensors, several limitations persist that hinder their widespread clinical application and commercial viability:

### 4.1. Technical Limitations

The fabrication of high-quality graphene remains a significant technical challenge. While graphene’s properties, such as high electron mobility and mechanical flexibility, are ideal for sensor applications, producing consistent, defect-free graphene at scale is difficult (see Figure 4c; note the combination of PI film, LBG, PDMS, and MXene, where each interface represents a potential point of failure that can compromise the entire device’s performance and longevity). Chemical Vapour Deposition (CVD), a common method for large-area synthesis, can introduce performance-degrading structural defects such as grain boundaries, wrinkles, and metallic catalyst residues [187]. These inconsistencies can lead to variations in sensor performance, particularly when detecting low-concentration analytes in biofluids. For instance, the functionalisation of graphene with bioreceptors, such as enzymes or aptamers, is essential for analyte specificity but can be unstable.

Moreover, the integration of graphene into flexible substrates for wearable applications often compromises its electrical properties, necessitating complex fabrication processes that are not yet cost-effective [188].

Overcoming the technical barriers to high-quality graphene synthesis requires scalable, reproducible fabrication methods. Advances in CVD optimisation, defect engineering, and alternative approaches such as laser-induced graphene (LIG) demonstrate considerable potential for producing uniform graphene sheets at lower cost. Stabilising functionalisation protocols for bioreceptor immobilisation (e.g., enzymes, aptamers) may significantly enhance sensor specificity and operational longevity. Effective integration with flexible substrates demands innovative materials and manufacturing strategies that preserve graphene’s electrical performance while maintaining mechanical compliance. These interdisciplinary developments are important for translating laboratory-based prototypes into commercially viable biosensing platforms.

### 4.2. Biological and Physiological Constraints

The biological variability of non-invasive biofluids poses a significant challenge to the reliability of graphene-based sensors. Graphene-based sensors frequently target biomarkers in biofluids such as sweat, tears, saliva or exhaled breath. Sweat, saliva, and tears exhibit considerable variation in composition due to factors such as hydration, diet, or physical activity, which complicates sensor calibration. For instance, glucose concentrations in sweat are typically 10–100 times lower than in blood, requiring sensors with exceptionally low detection limits [189,190]. Furthermore, analyte concentrations in these fluids can fluctuate rapidly, introducing time lags that affect real-time monitoring accuracy. A study by Torrente-Rodríguez et al. noted that sweat cortisol levels, while correlated with blood levels, exhibit delays in reflecting acute stress changes, reducing their utility for immediate diagnostics [93]. Additionally, while these biofluids are readily accessible and permit non-invasive sampling, their biochemical content often does not mirror blood concentrations accurately due to physiological transport mechanisms and compartmental kinetics. For instance, continuous glucose monitoring utilising biofluids must account for a physiological lag of around 15 min for tear and 20 min for sweat between blood and tear and sweat glucose levels [191], compromising real-time accuracy and critical hypoglycaemia detection [192]. Moreover, for gaseous biomarkers in exhaled breath, sensor arrays (‘e-noses’) face significant interference from high and variable background humidity, as well as cross-sensitivity to non-target ambient VOCs, which can confound the detection of specific disease biomarkers [193].

These sources underscore how biomarker choice and sampling medium significantly define sensor robustness. Though frequently claimed to be biocompatible, graphene-based nanomaterials exhibit nuanced and context-dependent biological interactions. Variants such as graphene oxide (GrO), rGrO or nanoplatelets elicit diverse cellular responses. For example, flake size, edge morphology, surface oxidation, dose and application site significantly influence cytotoxicity, oxidative stress, membrane disruption, inflammation and genotoxicity [194]. These effects, combined with a lack of standardised nanotoxicology protocols, restrict regulatory approval and long-term deployment in wearable health devices.

Further, biocompatibility has been unevenly studied: most assessments are in vitro, with limited long-term in vivo data or understanding of chronic environmental accumulation. As wearables aim for prolonged skin contact, continuous exposure to graphene nanoflakes risks sensitisation or an inflammatory response. Without exhaustive studies, reliance on such sensors may pose unforeseen health hazards over time. Additionally, the biocompatibility of graphene and its derivatives, such as GrO, remains a concern. While GrO is often cited for its improved dispersibility in biological environments, its long-term effects on skin or mucosal surfaces are not fully understood, raising questions about safety for continuous use [17].

Adaptive calibration algorithms, including machine learning approaches, can account for physiological lags, individual variability, and low analyte concentrations in biofluids. For breath-based sensing, improved sensor selectivity and humidity-compensation techniques are essential to mitigate cross-sensitivity and ambient interference. Long-term biocompatibility requires standardised in vivo protocols focused on chronic exposure, alongside evaluation of graphene-specific factors (e.g., flake size, surface oxidation, and morphology). Surface modifications (e.g., PEGylation or biocompatible polymer coatings) can reduce cytotoxicity and inflammatory responses. Future research should prioritise longitudinal clinical studies to validate safety and efficacy across diverse graphene derivatives. These efforts are important for advancing graphene-based sensors from experimental prototypes to clinically viable, non-invasive diagnostic tools.

### 4.3. Sensitivity, Selectivity and Matrix Interference

Graphene excels with high surface area and excellent electrical properties, but bare graphene is chemically inert; effective detection hinges upon functionalisation with enzymes, antibodies or aptamers. Polycyclic aromatic hydrocarbons (PAHs) have been shown to aid the dispersion of graphene in solvents, much like their use with single-walled carbon nanotubes (SWCNTs) [195]. Whilst larger PAHs exhibit stronger binding to graphene [196], similar to their interaction with SWCNTs [197], their limited solubility frequently restricts their practical application. Consequently, graphene is commonly modified, for instance via functionalisation, to suit real-world applications. While functionalisation enhances sensitivity, it also introduces variability in receptor immobilisation, potential drift, and limited long-term stability [198]. Continuous exposure to biofluids results in protein and salt deposition that reduces SNR and baseline drift, while the instability of biorecognition layers limits long-term operation [187]. This necessitates frequent recalibration, replacement or maintenance challenges that are antithetical to seamless, non-invasive wearables.

Enhancing long-term stability requires robust immobilisation methods (e.g., covalent bonding or cross-linked matrices) to minimise drift and improve bioreceptor retention. Anti-fouling coatings, notably zwitterionic polymers, can reduce protein and salt deposition, preserving signal fidelity. Regenerable interfaces and self-cleaning surfaces should be explored to reduce recalibration and maintenance requirements. Pilot studies in real-world conditions remain relevant to assess durability and performance. These strategies would enable the development of reliable, low-maintenance graphene-based sensors for seamless, non-invasive wearables.

**Figure 19:**
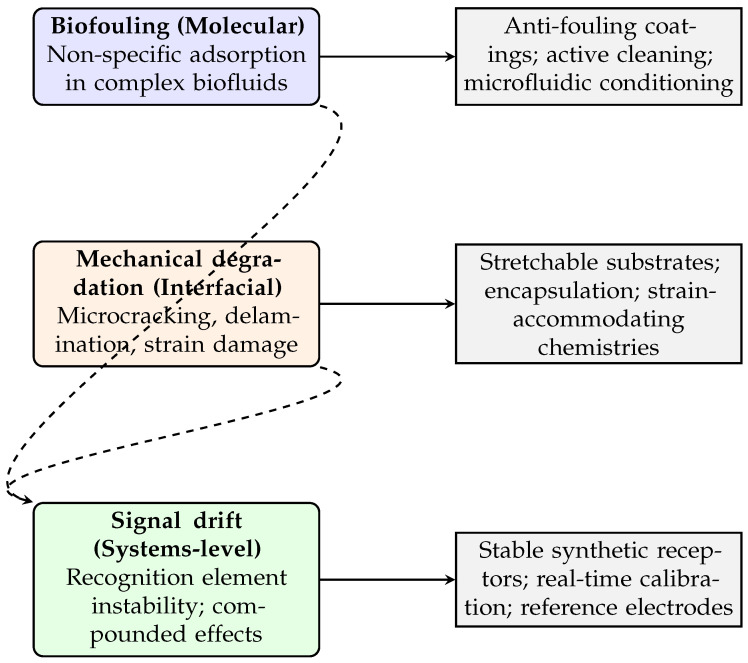
Schematic representation of the three principal failure modes in graphene-based wearable biosensors, mapped across molecular, interfacial, and systems-level domains. Arrows indicate causal relationships and compounding effects, while mitigation strategies are aligned to each mode to emphasise the necessity of coordinated, multi-scale intervention.

These recurring degradation pathways can be broadly categorised into three principal failure modes, namely biofouling, mechanical degradation, and signal drift, each arising from distinct underlying mechanisms and requiring tailored mitigation strategies. As summarised in Table 9 and illustrated schematically in Figure 19, these modes encompass molecular, interfacial, and systems-level challenges. Biofouling, prevalent in complex biofluids, requires both passive and active anti-fouling approaches to preserve sensor specificity. Mechanical degradation highlights the requirement for materials and structural designs capable of withstanding repeated strain without compromising conductivity or receptor integrity. Signal drift, often exacerbated by the first two modes, underscores the need for stable recognition elements and adaptive calibration algorithms. Addressing all three in a coordinated and integrated manner is essential for overcoming the most critical barriers to long-term, high-fidelity operation, thereby enabling the translation of graphene-based biosensors from laboratory prototypes to clinically robust, commercially viable wearable systems.

### 4.4. Practical and Commercial Challenges

Continuous monitoring requires a stable power source, and current graphene-based sensors often rely on external electronics, increasing device bulk and power demands. While energy harvesting technologies, such as triboelectric nanogenerators, have been proposed [199], they are not yet sufficiently efficient for practical use in wearables. The cost of producing graphene-based sensors remains a significant barrier to commercialisation. Although the cost of graphene has decreased, the processes for functionalising and integrating it into wearable platforms remain expensive. For example, laser-induced graphene (LIG) offers a cost-effective alternative to CVD, but its performance is less consistent, particularly for biochemical sensing applications [200]. Moreover, regulatory approval for medical devices requires extensive clinical validation, which is hindered by the lack of long-term studies on graphene-based sensors’ reliability and safety. A review by Liu et al. (2022) highlighted that most graphene-based sensors are still in the experimental stage [201]. Reproducibility is another major concern in scaling graphene biosensors. Variation in layer number, lateral flake dimensions, oxygen content, defect density and dopants leads to batch-to-batch performance inconsistency. Without standardised fabrication and characterisation protocols, commercial roll-out is impaired; calibration remains bespoke and costly.

Power demands may be mitigated through flexible energy harvesting systems (e.g., biofuel or hybrid generators integrated with the body). Manufacturing costs can be lowered by standardising laser-induced graphene and printing methods, combined with scalable transfer and post-processing for reproducibility. Establishing uniform fabrication and characterisation protocols will reduce variability, while long-term clinical validation through academic–industrial partnerships will accelerate regulatory approval. Together, these measures support the development of commercially viable graphene-based wearable biosensors.

### 4.5. Ethical and Societal Considerations

Ethical concerns, particularly around data privacy and accessibility, further complicate the adoption of graphene-based sensors. Wearable devices generate continuous streams of sensitive health data, necessitating robust encryption to prevent breaches. Graphene-based sensors, often integrated with wireless telemetry, are vulnerable to cyber threats, requiring advanced security measures that increase development costs [202]. Addressing these issues requires not only technological innovation but also policy frameworks to ensure equitable access and data protection.

Potential solutions include the adoption of robust encryption, blockchain-based data management, and privacy-by-design principles to mitigate cybersecurity risks. Early engagement with regulators, ethicists, and patient groups can help establish frameworks that safeguard both trust and equity, while the development of international standards for secure data handling will be essential for widespread adoption of wearable health technologies.

In conclusion, while graphene-based biosensors experience technical, biological, and commercial challenges, a range of emerging solutions—from scalable synthesis and hybrid material integration to advanced calibration, antifouling coatings, and secure data protocols—are being actively explored. By coupling materials innovation with clinical validation and ethical safeguards, researchers can move closer to realising robust, non-invasive graphene-based platforms for continuous health monitoring.

## 5. Conclusions and Future Perspectives

Graphene, a two-dimensional carbon nanomaterial, has emerged as a transformative platform for non-invasive biochemical sensing due to its exceptional electrical conductivity, large surface area, and intrinsic biocompatibility. By enabling biomarker detection through biofluids such as sweat, saliva, tears, and breath, graphene-based biosensors offer the prospect of near real-time health monitoring and early disease detection. However, despite remarkable progress, their clinical translation remains constrained by technical, biological, and practical challenges. These encompass issues of material reproducibility, device stability, sampling variability, and regulatory alignment. While graphene’s properties provide a robust foundation for biosensing, their effective deployment depends on resolving systemic challenges across four key domains:

- Translation Challenges: Most devices remain at the proof-of-concept stage, with limited evaluation under physiologically relevant conditions. Variations in biofluid composition, motion artefacts, and environmental interference impede reproducibility. Bridging this gap requires systematic in vivo validation, standardised biofluid sampling, and long-term stability assessment under real-world wear conditions.
- Clinical Benchmarks: For clinical adoption, graphene-based sensors must satisfy regulatory standards for accuracy, sensitivity, and specificity equivalent to gold-standard invasive assays. Achieving clinically relevant detection limits for glucose, lactate, cytokines, and tumour markers, while maintaining robustness across diverse populations, will require benchmark datasets and multi-site validation frameworks.
- Design Trade-offs: The same properties that make graphene appealing also introduce constraints. Pristine graphene offers high mobility but limited functionalisation, while graphene oxide improves bioreceptor immobilisation at the cost of conductivity. Increasing sensitivity often compromises mechanical integrity or biocompatibility. Future designs should explicitly balance these trade-offs through optimised hybrid materials and biofluid-specific architectures.
- Research Priorities: Key research directions include (i) scalable, reproducible synthesis of graphene derivatives; (ii) integration with wireless, low-power electronics for continuous data transmission; (iii) multiplexed platforms capable of detecting biochemical and biophysical cues; and (iv) longitudinal clinical studies to establish predictive value for disease monitoring.

Our benchmarking analysis (Table 8 and Figure 18) confirms that no single biofluid offers a universal sensing platform; each presents distinct advantages and constraints. Sweat sensors excel in wearability and dynamic tracking but face secretion variability and sensitivity–response trade-offs, where achieving low limits of detection may reduce response speed. Saliva sensors provide wide analyte coverage and excellent detection limits for proteins and viruses, yet are hindered by slow assay kinetics and limited validation under realistic oral conditions. Tear sensors achieve outstanding sensitivity through GFET designs but are constrained by minimal sample volumes and incomplete long-term biocompatibility data. Breath sensors remain early in development, with challenges in maintaining ppb-level sensitivity and selectivity under humid conditions.

Across biofluids, recurring issues such as biofouling, mechanical degradation, and signal drift (Table 9 and Figure 19) continue to undermine stability. The fragility of biorecognition elements and flexible interfaces under cyclic strain remains a major limitation. Addressing these will require innovations in antifouling coatings, strain-tolerant substrates, and durable synthetic receptors such as molecularly imprinted polymers and aptamers. Future efforts should shift from demonstrating sensitivity to ensuring operational resilience. Priorities include:

- Standardisation: Community-wide protocols for fabrication, functionalisation, and validation under physiologically relevant conditions.
- Multimodal Integration: Combining signals from multiple biofluids (e.g., sweat lactate and salivary cortisol) to deliver comprehensive physiological insights.
- Intelligent Systems: Machine learning-driven calibration, drift correction, and multimodal data fusion to address variability and temporal lag.
- Durability and Power Management: Extending operational lifespans (>1 month for disposables, >1 year for wearables) and developing self-sustaining or energy-efficient systems for autonomous operation.

In summary, graphene’s potential in non-invasive diagnostics is unequivocal, but its realisation depends on resolving the practical barriers of stability, reproducibility, and user-centred design. By confronting these challenges through sustained collaboration between materials scientists, engineers, and clinicians, graphene-based biosensors can progress from laboratory prototypes to reliable clinical technologies that advance proactive and personalised healthcare.

## Figures and Tables

**Table 1:** Comparative summary of key mechanistic attributes of Gr, GrO and rGrO.

Property	Gr	GrO	rGrO
Conductivity	Extremely high mobility; ideal for chemiresistive/FET detection (VOCs).	Very low; unsuitable for high-speed sensing without modification.	Intermediate; good for electrochemical biosensors.
Functionalisation potential	Limited covalent reactivity; relies on π–π stacking.	Very high; abundant surface functional groups enable dense enzyme/aptamer attachment.	Moderate; residual groups allow functionalisation and conductivity balance.
Mechanical properties	Exceptional (Young’s modulus ∼1 TPa, tensile strength ∼130 GPa).	Reduced strength; brittle in isolation.	Improved over GrO but not as strong as Gr.
Biocompatibility	Generally favourable; low cytotoxicity.	Hydrophilic and dispersible; may induce oxidative stress at high dose.	Balanced biocompatibility; less oxidative potential.
Application niches	Breath sensing; electrophysiology.	Tear/saliva sensing (protein/aptamer detection).	Sweat/saliva (electrochemical biosensing); flexible sensors.

**Table 2:** Comparison of properties of graphene, MXenes, silicene, and biochar.

Property/Application	Graphene	MXenes	Silicene	Biochar
Structure	Planar honeycomb lattice of carbon atoms	Layered transition metal carbides/nitrides with surface terminations (–O, –OH, –F)	Buckled honeycomb lattice of silicon atoms	Amorphous/graphitic porous carbon from biomass pyrolysis
Electronic Properties	Zero bandgap, high conductivity, Dirac fermions	Metallic conductivity, tunable surface chemistry	Small tunable bandgap (∼1.5 meV), high carrier mobility	Moderate conductivity; variable with precursor and processing
Surface Chemistry	High surface area, receptor immobilisation	Abundant functional groups, hydrophilic, excellent charge transfer	Reactive surface, analyte binding, functionalisation under development	Rich in O/N groups; enzyme immobilisation possible, but heterogeneous
Mechanical Properties	Flexible, strong, lightweight	Flexible in hydrogel form, stability issues	Predicted flexibility, less stable ambient	Robust and porous, limited flexibility in thin films
Biocompatibility	Generally good, widely studied	Good with polymers/hydrogels	Under investigation; stability/toxicity concerns	Variable; promising for processed forms, requires further validation
Applications in Biosensing	Glucose, lactate, cortisol, DNA/protein sensors	Electrochemical, wearable, enzymatic sensors	Early-stage; sensitive doped nanoribbons	Low-cost biosensors for glucose, dopamine, uric acid, antioxidants
Challenges	No intrinsic bandgap; synthesis challenges	Stability in aqueous/biological media; reproducibility	Ambient oxidation; immature synthesis	Lower conductivity, heterogeneity, batch variability

**Table 3:** Key characteristics and performance metrics of non-invasive sweat sensing devices, evaluated against defined benchmarks (sensitivity ≥10%; detection range (0.01–25 mM for metabolites or 10–100 mM for electrolytes); LOD < 10 μM; response time < 60 s; durability ≥ 8 h continuous use or ≥100 cycles). Unreported values (‘U’) are treated as not meeting the benchmark.

Sensing Material	Analyte Sample	Sensing Mechanism	Sensitivity (μA mM^−1^ cm^−2^)	Detection Range (mM)	LOD (mM)	Response Time (s)	Durability	Meets Benchmarks
Gr-PU-rGrO-PB/${\mathrm{LO}}_{x}$ [67]	Lactate	EC/patch	U	0.01–10.0	0.4	U	5 cycles	1/5
AuPt NPs/rGrO/chitosan-${\mathrm{GO}}_{x}$ [73]	Glc	AMP	48	0–2.4	0.005	20	192 h	5/5
PANI/TEGO/ PVA [74]	Glc	EC/patch	U	0.0002–10	0.0002	U	125 cycles	3/5
PBl/Au-doped Gr hybrid/${\mathrm{GO}}_{x}$ [75]	Glc	CV/patch	U	0.01–0.7	0.01	900	6 h	1/5
AgNC/3D LIGr/PtAuNP [76]	Glc	EC/patch	6.4	0–1.1	0.005	tens of seconds	408 h	5/5
${\mathrm{NH}}_{2}\mathrm{GP}/{\mathrm{Cu}}_{3}$ (BTC)^2^ [78]	Glc, Lac	EC/patch	Glc: 5360; Lac: 29	0.05–1.78; 0.05–22.6	0.00003; 0.005	5; 3	1200 h (for both)	5/5
SPE/PB/GrO-Ch/GO_x_ [79]	Glc, Lac	FIA	Glc: 8.2; Lac: 0.39	0.02–3.8; 1–50	6.7; 28	U U	35 m; 25 m	2/5
CNFs/CS-GrO [83]	Glc Urea	COL COL	U U	0.1–3; 30–180	0.1; 30	U	U	1/5
${\mathrm{Ti}}_{3}C_{2}T_{x}$ MXene /LBGr/PDMS [92]	CORT	IIS	U	$1.0\times 10^{-8}–1.0\times 10^{-4}$	$88\times 10^{-9}$	U	U	1/5
Graphene/ PI [93]	CORT	EC/patch	U	0.43–50.2 $\mathrm{ng}\;\cdot \;{\mathrm{mL}}^{-1}$	0.08 $\mathrm{ng}\;\cdot \;{\mathrm{mL}}^{-1}$	60	168 h	2/5
GNFET [96]	Cytokine	FE	U	$1.5\times 10^{-8}–2.5\times 10^{-4}$	$7.4\times 10^{-10}$	360	80 regenerative and 100 crumpling cycles	1/5
EGrFs/TPU, NMP [103]	${\mathrm{Na}}^{+}$	POT	58.3 mV/dec	0.1–100	0.0025	9.6	10,000 cycles	5/5
MOF/Gr [104]	${\mathrm{NH}}_{4}^{+}$	POT	59.23 mV/log	0.001–100	0.001	U	168 h	3/5
3D CVD Gr [105]	${\mathrm{Na}}^{+}$	POT	65.1 mV/dec	0.01–100	U	U	125 h	1/5
Paper-based ISE (rGrO/ FAS) [106]	$K^{+}$ ${\mathrm{Cl}}^{-}$ pH ${\mathrm{Na}}^{+}$	POT	57.0 mV/dec 56.7 mV/dec 56 mV/dec 55.7 mV/dec	$1.0\times 10^{-3}–1.0\times 10^{2}$	6.5 61.4 6.91 49.5	U	12 h	1/5
GFET [107]	L-Cys	FET	U	0–4.8	0.00022	U	100 cycles	3/5

EC = Electrochemical, AMP = Amperometric, Lac= Lactate, U = Unknown.

**Table 4:** Key characteristics and performance metrics of non-invasive saliva sensing devices (Part 1/2), evaluated against defined benchmarks. Benchmarks: sensitivity (≥10% signal change); detection range (pM–nM for proteins/viruses or relevant clinical range for metabolites); LOD (<100 pg/mL for proteins/viruses or <1 μM for metabolites); response time (<5 min); and durability (>30 days storage stability). Unreported values (‘U’) are treated as not meeting the benchmark.

Sensing Material	Analyte Sample	Sensing Mechanism	Sensitivity (μA mM^−1^ cm^−2^)	Detection Range	LOD (Lowest Tested)	Response Time (min)	Durability (Day)	Meets Benchmarks
GrO-Au/FBG [115]	COVID-19 virus	SPR	0.4250 × 10^−8^ nm/virus number	1.6 × 10^3^–1.2 × 10^8^ copies·mL^−1^	1.6 × 10^3^ copies·mL^−1^	0.17	U	3/5
DNA-apt-GFET [116]	SARS-CoV-2 virus	EC	U	0–30 nM (S protein); 0–15 nM (N protein)	1.28 PFU·mL^−1^; 1.45 PFU·mL^−1^	20	U	1/5
DGTFET [117]	IFN-γ; IL-6; TNF-α	EC	U	1 nM–10 pM; 10–200 pM; 10–200 pM	476 fM; 611 fM; 608 fM	7	U	2/5
rGrO-FET [120]	HPV-16 E7	EC	U	30–1000 nM	1.75 nM	U	30	2/5
rGrO/MoS_2_ /GCE [121]	HPV-16 L1	DPV	U	0.2–2 ng·mL^−1^ (3.5 pM–35.3 pM)	0.1 ng·mL^−1^ (1.75 pM)	40	30	2/5
TrGrO [122]	H1N1	EC	U	0–10,000 PFU·mL^−1^	33.11 PFU·mL^−1^	U	14	2/5
LBA-Gr-GCE [123]	LYS	EC	U	0.01–0.5 pM·L^−1^	6 fM·L^−1^	23	200 cycles	2/5
GrO/ssDNA [124]	LYS	FL	U	1.6–14.4μL	1.4 μ L	20	U	1/5
AuNPs/Ppy-COOH/Gr-SPE [127]	PVD	EC/DPV	U	1–100 μM/L	0.33 μM/L	U	8 tests	2/5
PtNP@Gr /SPCE [130]	COT	EC/CV	1.89μA/ decade^−1^	1–100 ${\mathrm{nML}}^{-1}$	0.33 ${\mathrm{nML}}^{-1}$	12	U	3/5
MX/Gr [131]	NIC	EC/DPV; EC/AMP	3.5; 0.527	1–55 μM; 30–600 nM	290 nM; 0.28 nM	U	40	4/5
KT-MIM/MOFs @Gr/SPE [133]	KET	DPV	U	1 × 10^−10^–4 × 10^−5^ M·L^−1^	4.0 × 10^−11^ M·L^−1^	5	60 uses or 60 days	4/5
TiO_2_-GrO/CPE [134]	BEN; ANT	EC/SWV	U	1μM–1.0 mM; 12 nM–80 μM	0.25 μM; 3 nM	U	30	2/5
N-prGrO-(py-PEG/ PyCOOH)/GCE [136]	cTnI	EC/DPV	41 μA cm^−2^·decade^−1^	0.001–100 ng·mL^−1^	1 pg·mL^−1^	30	10 cycles; 30 days	4/5
anti-Mb-Ab/d-BSA/rGrO [137]	Mb	EC/EIS	U	5 pM–10 nM	2.37 pM	30	U	2/5
Lg-GFET [138]	CORT	EC	U	0.08–800 nM	U	30	U	1/5
Gr/PPy [139]	CORT	EC/patch	U	0.5–5 ng·mL^−1^	0.5 ng·mL^−1^	U	U	1/5
GrP/PS67-b-PAA_27_ [140]	CORT	EC	50 Ω (pg mL^−1^)^−1^	3 pg·mL^−1^–10 μg·mL^−1^	3 pg·mL^−1^	12	28	3/5

EC = Electrochemical, CR = Chemiresistive, U = Unknown, 1 month = 30 days.

**Table 5:** Key characteristics and performance metrics of non-invasive saliva sensing devices (Part 2/2), evaluated against the same benchmarks as defined in Table 4. Benchmarks: sensitivity (≥10% signal change); detection range (pM–nM or relevant clinical range); LOD (<100 pg/mL for proteins/viruses or <1 μM for metabolites); response time (<5 min); and durability (>30 days storage stability). Unreported values (‘U’) are treated as not meeting the benchmark.

Sensing Material	Analyte Sample	Sensing Mechanism	Sensitivity (μA mM^−1^ cm^−2^)	Detection Range	LOD	Response Time (min)	Durability (Day)	Benchmarks
Gr/PS67-b-PAA_27_ [141]	CORT	EC	U	0.001– 10 ng mL^−1^	0.87 pg mL^−1^	12	42	3/5
c-Mab-rGrO/ITO/glass [142]	CORT	EC/CR	U	1–10 ng mL^−1^	27.6 pM	U	several months	3/5
Ab/d-BSA/rGrO/Qz [143]	CORT	EC/EIS	U	10–10,000 pM	10 pM	30	U	2/5
GrO-CS/GSPE [144]	5-HT	EC/DPV	0.05 μA$\mu M^{-1}$	0.01–100 μM	3.2 nM	30	28	2/5
AuNP/rGrO /SPE [145]	Trp	EC/DPV	U	0.5–500 μM·L^−1^	0.39 μM·L^−1^	U	U	1/5
Gr-PLA [146]	UA	EC/BIA-MPA	0.1332 μA·L·μM^−1^	0.5–250 μM·L^−1^	0.02 μM·L^−1^			
		EC/DPV	0.1723 μA·L·μM^−1^	10–70 μM·L^−1^	0.5 μM.L^−1^			
	NO_2_^−^	EC/BIA-MPA	0.0922 μA·L·μM^−1^	0.5–250 μM·L^−1^	0.03 μM·L^−1^	U	15 measurements	1/5
		EC/DPV	0.0031 μA·L·μM^−1^	50–1300 μM·L^−1^	30 μM·L^−1^			
	Glc	AMP	U	0.50– ∼6.30 mM·L^−1^	15 μM·L^−1^			
CuNPs/LIGr [147]	Glc	EC	2665	0.03–4.5 mM	0.023 µM	0.083	35	4/5
GrO-μPAD [148]	Glc	COL	U	0–∼1 mM	0.02 mM	1	U	2/5
Cu_2_O NC/Gr [149]	Glc	EC	36.4	0.002–17.1 mM	0.23 μM	1	180	4/5
Au/rGrO-Ti_3_C_2_ [150]	Glc	EC	355	10 µM–21 mM	3.1 µM	0.083	10	2/5
PEDOT-GrO/ITO [151]	UA	EC/CV	U	2–1000 μM	0.75 μM	1	10	2/5
GFET [152]	IL-6	POT	U	0.05–0.84 nM	12.2 pM	6.67	U	2/5
GFET [153]	CA1	EC	65.4 mV·decade^−1^	330 fM– 3 nM	330 fM	60	U	3/5
rGrO/ITO [155]	CYFRA-21-1	EC/DPV	0.756 mA mL·ng^−1^	2–22 ng·mL^−1^	0.122 ng·mL^−1^	16	56	3/5
BSA/anti-CYFRA21/ APTES/nHfO_2_ @rGrO/ITO [156]	CYFRA-21-1	EC/DPV	18.24 µA mL·ng^−1^	0–30 ng·mL^−1^	0.16 ng·mL^−1^	15	40	3/5
AuNPs-rGrO [157]	IL8	EC	U	0.0005–4 ng mL^−1^	72.73 pg·mL^−1^	9	84	3/5
ZnO-rGrO [158]	IL8	EC	12.46 µA·mL·ng^−1^	100 fg·mL^−1^– 5 ng·mL^−1^	51.53 pg·mL^−1^	10	70	4/5
GrP-PS67-b-PAA27-Au [159]	PSA	EC/CR	0.875 $\Omega \;{\mathrm{fg}}^{-1}\;\mathrm{mL}$	0.0001–100 ng mL^−1^	40 fg·mL^−1^	4	56	5/5
MWCNT/His-rGrO [160]	PSA	EC/DPV	U	0.01–20,000 pg mL^−1^	2.8 fg·mL^−1^	25	28	2/5

**Table 6:** Key characteristics and performance metrics of non-invasive tear sensing devices, evaluated against defined benchmarks (sensitivity ≥10% signal change across 0.1–0.9mM for glucose or the picomolar range for cytokines; detection range 0.1–0.9mM for glucose or $10^{-12}$–$10^{-9}\;M$ for cytokines; LOD <5μM for glucose or <0.1nM for cytokines; response time < 30 s; durability > 24 h). Unreported values (‘U’) are treated as not meeting the benchmark.

Sensing Material	Analyte Sample	Sensing Mechanism	Detection Sensitivity	Detection Range	LOD	Response Time (s)	Durability	Meets Benchmarks
Gr-AgNW [46]	Glc	EC	U	0.001–10 mM	0.4 μM	U	Stable after 5000 cycles; enzyme activity retained 24 h in solution	3/5
Gr/CAT /${\mathrm{GO}}_{x}$ [174]	Glc	EC	22.72% ${\left(\mathrm{mM}\right)}^{-1}$	0.1–0.9 mM	12.57 μM	∼1.3	Stable for 48 h in artificial tears; negligible degradation after 5000 cycles of stretching	4/5
GFET/ PMMA /PASE [175]	Cytokines TNF-α	FE	U	0.03–500 nM	2.75 pM	∼420	Consistent response after 100% tensile strain	2/5
	Cytokines IFN-γ	FE	U	0.03–500 nM	2.89 pM	U	(Same as TNF-α)	2/5
GFET/IgG /AgNWs [176]	MMP-9	FE	11.1 ng ${\mathrm{mL}}^{-1}$ per 1% $\Delta I_{d}$	1–500 ng ${\mathrm{mL}}^{-1}$	0.74 ng ${\mathrm{mL}}^{-1}$	∼2.5	Stable after 16 days of accelerated aging (≈1 year storage period)	3/5
PEDOT-Gr [177]	DA	AMP	12.9 μA ${\mathrm{mM}}^{-1}{\mathrm{cm}}^{-2}$	0–70 μM	101 nM	U	High long-term stability; ∼15% sensitivity loss after 1 month of storage at 4 °C	1/5
GFET [107]	L-Cys	FE	U	0–4800 μM	0.02 μM in undiluted human sweat; 0.043 μM in artificial tears	U	Consistent electrical and mechanical properties after 100 bending/folding/shrinking cycles	2/5

EC = Electrochemical, FE = Field Effect, DA = Dopamine, AMP = Amperometric, U = Unknown.

**Table 7:** Key characteristics and performance metrics of non-invasive breath sensing devices, evaluated against defined benchmarks (sensitivity ≥10%, detection range spanning clinically relevant concentrations (ppb to low-ppm), LOD < 100 ppb/0.1 ppm, response time <60s, durability under ≥80% RH). Unreported values (‘U’) are treated as not meeting the benchmark.

Sensing Material	Analyte Sample	Sensing Mechanism	Detection Sensitivity	Detection Range (ppm)	LOD (Lowest Tested, ppm)	Response Time (s)	Durability	Meets Benchmarks
Porphyrin-rGO [182]	Acetone, ${\mathrm{NH}}_{3}$	EC	Unique VOC patterns	25–100	25	∼seconds	U	2/5
rGrO/OA [183]	EtOH, 2-ethylhexanol, nonanal	CR	High sensitivity at 25 ppm	25–125	25	61–200	Stable response	2/5
CS-rGrO [184]	Acetone	CR	27.89% response at 10 ppm	0–10	U	U	5 weeks	2/5
PGr-${\mathrm{SnO}}_{2}$ [185]	Ethanol	CR	>35% response	0.5–2	0.5	∼50	U	
	Acetone		>35% response	0.5–4	0.5	∼50	U	
	NO		>5% response	0.01–0.1	0.01	∼50	U	3/5
	CO		>5% response	1–5	1	∼50	U	
PGr-Cu BTC [186]	MeOH, Chloroform	CR	Highest sensitivity for Chloroform (value not provided)	2.82–22.6	2.82	∼seconds	U	2/5

EC = Electrochemical, CR = Chemiresistive, U = Unknown.

**Table 8:** Cross-biofluid benchmarking summary of non-invasive graphene-based sensing devices, aggregating performance across sweat (Table 3), saliva (Table 4 and Table 5), tear (Table 6), and breath (Table 7). Columns show the distribution of devices by benchmarks met (out of 5), with both counts and percentages, alongside average scores (±SD) and key strengths/limitations derived from biofluid-specific evaluations.

Sensors	1/5	2/5	3/5	4/5	5/5	Avg. ± SD	Strengths	Limitations
Sweat (16)	7 (44%)	2 (13%)	3 (19%)	0 (0%)	4 (25%)	2.5 ± 1.6	High durability; rapid response; clinically relevant ranges; multiple perfect-score devices; excellent sensitivity; scalable wearable designs; broad analyte compatibility	Inconsistent LOD; high performance variance; many devices at low maturity level; inconsistent response time reporting; trade-offs: sensitivity vs. responsiveness; durability issues under real sweat conditions;
Saliva (37)	6 (16%)	15 (41%)	9 (24%)	6 (16%)	1 (3%)	2.5 ± 1.0	Exceptional LOD; broad analyte diversity; many devices meet high benchmarks; hybrid nanocomposites enable balanced sensitivity and stability; large device pool with miniaturised designs	Slow response; inconsistent sensitivity reporting; limited real-world stability data; sensitivity–speed trade-off; durability gaps; variable performance metrics; heterogeneous reporting; missing real-matrix validation
Tear (6)	1 (17%)	3 (50%)	1 (17%)	1 (17%)	0 (0%)	2.3 ± 0.9	Ultra-low LOD; rapid response; good durability; suitable for contact-lens integration; good mechanical stability with direct corneal access	Small evidence base; sensitivity–speed–durability trade-offs; few devices fully validated; limited ocular biocompatibility data; incomplete long-term stability testing; limited multiplexing exploration
Breath (5)	0 (0%)	4 (80%)	1 (20%)	0 (0%)	0 (0%)	2.2 ± 0.8	Fast response; clinically relevant VOC detection; high sensitivity; rapid chemiresistive sensor responses; real-time VOC monitoring; unique exhaled breath profiles; strong diagnostic potential	Poor LOD profile and durability in high humidity; low performance variance; material challenges for humidity-resistant selectivity; sparse real-breath validation data; translational immaturity

Note: Benchmarks are biofluid-tailored: sweat—LOD < 10 µM, response time < 60 s, durability > 8 h; saliva—LOD < 100 pg/mL (proteins) or <1 µM (metabolites), response time < 5 min, stability > 30 days; tear—LOD < 15 µM (glucose) or <1 nM (cytokines), response time < 30 s, durability > 12 h; Breath—LOD < 100 ppb, response time < 60 s, stability at ≥80% RH. “U” = unknown/unreported, scored as fail, intended to prevent optimistic bias. Percentages = (devices in tier/total devices) × 100. Avg. Percentages are rounded. Benchmark score = mean ± SD of per-device benchmark compliance (1–5 scale). SD reflects variability in benchmark scores across devices within each biofluid.

**Table 9:** Summary of principal failure modes in graphene-based wearable biosensors, their underlying mechanisms, performance impacts, and representative mitigation strategies. The modes encompass molecular (biofouling), interfacial (mechanical degradation), and systems-level (signal drift) challenges, highlighting the need for integrated solutions to achieve long-term, high-fidelity bioprofiling.

Failure Mode	Underlying Mechanism	Impact on Performance	Representative Mitigation Strategies
Biofouling (molecular)	Non-specific adsorption of proteins, cells, and macromolecules from complex biofluids onto the graphene surface	Reduced sensitivity and specificity; baseline signal drift	Surface functionalisation with anti-fouling coatings (e.g., PEG, zwitterionic polymers); active electrochemical cleaning; microfluidic sample conditioning
Mechanical degradation (interfacial)	Microcracking, delamination, or strain-induced disruption of conductive pathways and receptor anchoring sites	Loss of conductivity; reduced binding efficiency; device failure under repeated deformation	Use of stretchable substrates and serpentine interconnects; encapsulation layers; strain-accommodating receptor immobilisation chemistries
Signal drift (systems-level)	Instability of recognition elements; environmental fluctuations; compounded effects of biofouling and mechanical degradation	Gradual baseline shift; reduced accuracy and reliability over time	Incorporation of stable synthetic receptors (e.g., aptamers, MIPs); real-time calibration algorithms; reference electrode integration

## Data Availability

No new data were created or analysed in this study. Data sharing is not applicable to this article.

## Abbreviations

The following abbreviations are used in this manuscript:

AgNWs	Silver Nanowires
APTES	3-Aminopropyl triethoxy silane
AuPt NPs	Gold and Platinum Alloy Nanoparticles
BSA	Bovine Serum Albumin
CA1	Carbonic Anhydrase 1
CAT	Catalase
CEA	Carcinoembryonic Antigen
CFTR	Cystic Fibrosis Transmembrane Conductance Regulator
CO	Carbon Monoxide
CR	Chemiresistive
CS	Chitosan
CV	Cyclic Voltammetry
CVD	Chemical Vapour deposition
cTnI	Cardiac Troponin I
CYFRA-21-1	Cytokeratin 19 Fragment
DA	Dopamine
DNA	Deoxyribonucleic Acid
DPV	Differential Pulse Voltammetry
EC	Electrochemical
ECD	Electrochemical Deposition
ECG	Electrocardiograms
EEG	Electroencephalograms
ELISA	Enzyme-Linked Immunosorbent Assay
EMG	Electromyograms
EPD	Electrophoretic Deposition
EtOH	Ethanol
GCE	Glassy Carbon Electrode
GFET	Graphene Field-Effect Transistor
GNFET	Graphene-Nafion Field-Effect Transistor
GOx	Glucose Oxidase
Gr	Graphene
GrO	Graphene Oxide
GrP	Graphene on Paper
HIS-rGrO	L-Histidine-Modified Reduced Graphene Oxide
HWE	Hybrid Working Electrode
IDO	Indium Tin Oxide
IFN-γ	Interferon Gamma
IgG	Immunoglobulin G
IL-6	Interleukin-6
IL-8	Interleukin-8
IoT	Internet of Things
ITO	Indium Tin Oxide
LBGr	Laser-Burned Graphene
LDH	Lactate Dehydrogenase
LDT	Low Detection Threshold
LIG	Laser-Induced Graphene
LOD	Limit of Detection
LOQ	Quantification Limit
LOx	Lactate Oxidase
MeOH	Methanol
MMP-9	Matrix Metalloproteinase-9
MOF	Metal–Organic Framework
MOFs	Metal–Organic Frameworks
MWCNT	Multiwalled Carbon Nanotube
Na^+^	Sodium Ion
NH^+^	Ammonium Ion
NH_3_	Ammonia
NMO	Nanostructured Metal Oxide
nHfO_2_	Nanostructured Hafnium Oxide
NO	Nitric Oxide
NTA	Nanoparticle Tracking Analysis
OA	Octylamine
OS	Oxidative Stress
OSI	Ocular Scatter Index
PAH	Polycyclic Aromatic Hydrocarbon
PANI	Polyaniline
PASE	1-Pyrenebutanoic Acid Succinimidyl Ester
PBS	Phosphate Buffer Saline
PDMS	Polydimethylsiloxane
PEDOT-Gr	Poly(3,4-ethylene dioxythiophene)-Graphene
PI	Polyimide
PMMA	Polymethyl Methacrylate
POC	Point-of-Care
ppb	Parts Per Billion
ppm	Parts Per Million
PGr	Porous Graphene
PGr-Cu BTC	Pristine Graphene-Copper Benzene-1,3,5-tricarboxylate
PGr-SnO_2_	Pristine Graphene-doped Tin Oxide
PGr-UiO 66	Pristine Graphene-Zirconium 1,4-dicarboxybenzene
PGr-ZIF 8	Pristine Graphene-2-methylimidazole Zinc Salt
PS67-b-PAA27	Polystyrene-block-poly(acrylic acid)
PSA	Prostate-Specific Antigen
PTFE	Polytetrafluoroethylene
PVA	Polyvinyl Alcohol
rGrO	Reduced Graphene Oxide
RNA	Ribonucleic Acid
RLC	Resistor-Inductor-Capacitor
RSD	Relative Standard Deviation
SNR	Signal-to-Noise Ratio
SWCNTs	Single Walled Carbon Nanotubes
TEGrO	Thermally Exfoliated Reduced Graphene Oxide
THF	Tetrahydrofuran
TNF-α	Tumor Necrosis Factor-alpha
UiO 66	University of Oslo Framework 66
VOC	Volatile Organic Compound
VPP	Vapour Phase Polymerisation
WO_3_/Au/WO_3_	Tungsten Trioxide/Gold/Tungsten Trioxide
WSAS	Wireless Self-powered Acetone Sensor
ZIF 8	Zeolitic Imidazolate Framework 8
ZnO	Zinc Oxide
